# JMJD3 aids in reprogramming of bone marrow progenitor cells to hepatic phenotype through epigenetic activation of hepatic transcription factors

**DOI:** 10.1371/journal.pone.0173977

**Published:** 2017-03-22

**Authors:** Veena Kochat, Zaffar Equbal, Prakash Baligar, Vikash Kumar, Madhulika Srivastava, Asok Mukhopadhyay

**Affiliations:** 1 Stem Cell Biology Laboratory, National Institute of Immunology, New Delhi, India; 2 Epigenetic Regulation Research Laboratory, National Institute of Immunology, New Delhi, India; Universita degli Studi Di Cagliari, ITALY

## Abstract

The strictly regulated unidirectional differentiation program in some somatic stem/progenitor cells has been found to be modified in the ectopic site (tissue) undergoing regeneration. In these cases, the lineage barrier is crossed by either heterotypic cell fusion or direct differentiation. Though studies have shown the role of coordinated genetic and epigenetic mechanisms in cellular development and differentiation, how the lineage fate of adult bone marrow progenitor cells (BMPCs) is reprogrammed during liver regeneration and whether this lineage switch is stably maintained are not clearly understood. In the present study, we wanted to decipher genetic and epigenetic mechanisms that involve in lineage reprogramming of BMPCs into hepatocyte-like cells. Here we report dynamic transcriptional change during cellular reprogramming of BMPCs to hepatocytes and dissect the epigenetic switch mechanism of BM cell-mediated liver regeneration after acute injury. Genome-wide gene expression analysis in BM-derived hepatocytes, isolated after 1 month and 5 months of transplantation, showed induction of hepatic transcriptional program and diminishing of donor signatures over the time. The transcriptional reprogramming of BM-derived cells was found to be the result of enrichment of activating marks (H3K4me3 and H3K9Ac) and loss of repressive marks (H3K27me3 and H3K9me3) at the promoters of hepatic transcription factors (HTFs). Further analyses showed that BMPCs possess bivalent histone marks (H3K4me3 and H3K27me3) at the promoters of crucial HTFs. H3K27 methylation dynamics at the HTFs was antagonistically regulated by EZH2 and JMJD3. Preliminary evidence suggests a role of JMJD3 in removal of H3K27me3 mark from promoters of HTFs, thus activating epigenetically poised hepatic genes in BMPCs prior to partial nuclear reprogramming. The importance of JMJD3 in reprogramming of BMPCs to hepatic phenotype was confirmed by inhibiting catalytic function of the enzyme using small molecule GSK-J4. Our results propose a potential role of JMJD3 in lineage conversion of BM cells into hepatic lineage.

## Introduction

Lineage commitment and differentiation of somatic cells have been considered as unidirectional events. However, reports on BM cells’ plasticity, generation of induced pluripotent stem cells (iPSCs) from somatic cells, and direct reprogramming of fibroblasts to other lineages have led to a paradigm shift in this belief [1–5]. Adult BM cells were shown to convert into different lineages, depending on the cellular competency for differentiation and the cues to which they have been exposed [6–8]. BM cells were shown to undergo lineage conversion to hepatocytes through either direct differentiation or cell fusion [6, 9–16]. Using *Cre-LoxP* system in lethally irradiated *FAH*^*-/-*^ transgenic mouse, with high selection pressure for the replacement of hepatocytes, it was confirmed that BM-derived hepatocytes were the product of fusion heterokaryons, generated by polyploidization followed by ploidy reduction [17]. Whereas in other acute injury models, direct differentiation of BM cells into hepatocytes was proposed [11,12]. Irrespective of the pathways followed, nuclear reprogramming of cells was considered inevitable for the fate change event. It was observed that in case of somatic cell nuclear transfer or transcription factors-induced fate change of somatic cells, nuclear reprogramming is associated with sequential silencing of critical genes in the starting cells followed by activation of genes leading to the new fate [18].

The hallmark of nuclear reprogramming is erasure of original epigenetic identity followed by establishment of new epigenetic signatures. Such coordinated epigenetic modifications have an important role in lineage specification, stable gene expression and maintenance of cellular phenotype. Certain marks like acetylation and methylation are present in the N-terminal chains of the histones that constitute the nucleosome which regulate transcription by altering the compactness of chromatin and accessibility of specific genomic regions to transcription factors. Post-translational modifications of histone side chain residues [19] and methylation of DNA [20] at regulatory regions of genes contribute to the stability of chromatin structure that control the expression pattern of lineage specific genes. How the unique lineage gene expression pattern of uncommitted BM cells is refashioned to a hepatic expression program and whether this change is stably maintained *in vivo* is not clearly understood. The only study on gene expression analysis of BM-derived hepatocytes through genome-wide expression analysis described the formation of intermediate cells that differ from the hematopoietic and hepatocyte lineages, but no epigenetic modifications in these cells were investigated [21]. We have shown earlier that uncommitted syngeneic or allogeneic donor BM progenitor cells (Lin^-^) are involved in liver regeneration by engraftment and lineage conversion in acute liver injury model of hemophilia A mouse leading to therapeutic correction [12, 22]. Lin^-^ cells are composed of both Lin^-^CD45^+^ and Lin^-^CD45^-^ fractions, both fractions are important for functional regeneration of liver parenchyma and non-parenchyma, without that hemophilic mouse would not survive. Since BM cells have been used in many clinical trials for the treatment of liver diseases, it is imperative to understand the molecular and functional basis of liver regeneration by BM-derived cells. Male hemophilia A (HA) mice do not synthesize functional factor VIII, so die as a result of blood loss. Our previous studies confirmed the functional improvement of mice as donor derived cells not only involved in the repairmen of damage liver but also they synthesize active factor VIII [12,22]. We hypothesize that erasure of original epigenetic signatures followed by establishment of new epigenetic marks at lineage specific gene promoters by chromatin modifying enzymes, induced in the injured liver micro-environment, direct the change in transcriptional program of uncommitted BM cells in hepatic gene expression program. To test our hypothesis we performed comprehensive transcriptional profiling to analyze change in gene expression that accompany reprogramming of BMPCs to hepatocyte-like cells during liver regeneration in acute liver injury model of HA mice. We also determined the change in histone modifications at lineage specific gene promoters and the role of chromatin modifying enzymes in facilitating the induction and maintenance of nuclear reprogramming in BM-derived hepatic cells. Our findings, for the first time, provide new insights into chromatin dynamics that regulate the transcriptional reprogramming mechanisms occur in BMPCs during commitment to hepatocyte-like lineage.

## Materials and methods

### Isolation and transplantation of BM cells

Acute liver injury was induced in six to eight weeks old hemophilia A (HA) male mice [B6;129S4-F8^tm1Kaz^/J] by administering acetaminophen (500 mg/kg body weight) through intra-peritoneal injection. Bone marrow cells were isolated from healthy femurs and tibiae of 6–8 weeks old female eGFP-expressing Bl6/J [C57Bl6/J-Tg(UBCGFP) 30Scha/J] mice from which Lin^-^ cells were separated by MACS following a negative selection method using mouse hematopoietic progenitor cell enrichment kit (Stem Cell Technologies, Vancouver, British Columbia, Canada) which consist of antibody cocktail containing CD5, CD11b, CD19, CD45R, 7–4, Ly-6G/C (Gr-1) and Ter119 antibodies. Before harvesting bone marrow cells, euthanasia of mice was carried out by inhaling excess CO_2_. Within 30 h of liver injury, 0.25 × 10^6^ Lin^-^ eGFP cells were transplanted into HA mice through tail vein. CD45^+^ and CD45^-^ cells were isolated from the Lin^-^ eGFP cells using FACS Aria III (BD Biosciences, San Diego, CA) and 0.125 × 10^6^ cells were transplanted in each mouse. Animals were procured from The Jackson Laboratories; all experiments were conducted as per procedures approved by the Institutional Animal Ethics Committee, National Institute of Immunology, New Delhi.

### Isolation of hepatocytes

Mice were anesthetized by intraperitoneal injection of ketamine (100 mg/kg) and xylazine (10 mg/kg). In step I, the liver was perfused with Ca^2+^/Mg^2+^ ion-free HBSS buffer (25 ml) containing 0.5 mM EGTA, 25 mM HEPES at 37°C for 15 min. In the step II, 25 ml of the HBSS buffer, lacking EGTA, but containing 0.15 mg/ml collagenase I and 5 mM CaCl_2_ was used for perfusion. The liver capsule was incised and single-cell suspension was prepared in RPMI-1640 containing 10% FBS and separated through 100 μm cell strainer. Cell suspension was centrifuged at 50*g* for 5 minutes to isolate hepatocytes from non-parenchyma cells. Donor BM-derived hepatocytes were isolated from single cell suspension on the basis of eGFP-expression using FACS Aria III (BD Biosciences, San Diego, CA). The sorted cells were confirmed as hepatocytes by staining with anti-albumin antibody. These BM-derived hepatocytes were used for microarray and gene expression analysis, FISH and ChIP.

### Immunostaining of cells/tissues

The cells or tissue sections fixed with 4% para-formaldehyde were permeabilized with 0.1% saponin in PBS for 30 minutes and blocked with 3% gelatin solution for 1 hour. Cells/tissues were incubated for 1 hour at room temperature or 10 hours at 4°C with primary antibodies. After washing, samples were counter-stained with secondary antibodies for 1 hour. The primary antibodies used for immuno-histocmical/ immuno-cytochemical analyis were APC conjugated anti-CD45 (eBioscience, San Diego, CA); anti-GFP (Clontech-Takara Bio Company, Kyoto, Japan); anti-albumin (Bethyl Laboratories, Montgomery, USA); anti-HNF4α, anti-E-cadherin, anti-CK18 (Santa Cruz Biotechnology, Santa Cruz, CA); anti-JMJD3, anti-EZH2, anti-H3K27me3 (Abcam, Cambridge, UK). Secondary antibodies used were donkey anti-mouse Alexa fluor 488, donkey anti-goat Alexa fluor 594, donkey anti-rabbit Alexa fluor 594, donkey anti-goat Alexa fluor 488 (Molecular Probes Inc., Eugene, USA). Washed cells/sections were treated with 4’,6-diamidino-2-phenylindole, DAPI (10 μg/ml) and mounted using ProLongR anti-fade (Molecular Probes Inc., Eugene, Oregon, USA). Immuno-stained cells/sections were observed under Olympus fluorescence microscope (Model IX51) and images were acquired by DP70 digital camera (Tokyo, Japan) using Image Pro software (Media Cybernetics, Inc., Rockville, MD). High-magnification images were taken with a Zeiss LSM 510 META confocal laser-scanning microscope (Carl Zeiss, Oberkochen, Germany) using a Plan-Apochromat 63×/1.4 oil objective with LSM 510 software for image acquisition and Zeiss LSM Image browser version 4.2.0.121 for processing.

### Fluorescent in situ hybridization for X,Y chromosome

Sorted hepatocytes were treated with 0.56% KCl at 37°C for 45 minutes, fixed with Methanol:Acetic acid (3:1) and dropped on chilled slides to obtain a uniform spread. Cells were fixed with 70% Acetic acid and dehydrated in series of alcohol (70%, 90% and 100%). Cells were digested with 0.1 mg/ml pepsin/0.01N HCl for 30 minutes at 37°C. Nuclei were fixed with 1% PFA, dehydrated and denatured in 70% formamide/2×SSC at 76°C for 6 minutes. Poseidon^™^ RAB9B (XqF1) / WC Y mouse probes were denatured at 90°C for 10 minutes and added to sample and sealed. Hybridization was carried out at 37°C for 12–18 hours in a humidified chamber. Slides were washed in 0.4 x SSC/0.3% NP-40 at 72°C followed by 2 x SSC/0.1% NP-40 at RT. Slides were dehydrated and nuclei were counterstained with DAPI at 10 μg/ml concentration. Samples were mounted with ProLongR anti-fade and scored using an upright Olympus BX51 fluorescence microscope (1000×). CytoVisionTM and GenusTM imaging softwares (Applied Imaging, CA) were used to capture the images.

### Relative quantification of genes by real-time PCR

Total RNA was isolated from cells using TRIZOL^®^ Reagent (Invitrogen, Carlsbad, CA, USA) or RNAqueous Micro RNA isolation kit (Ambion^®^ Life Technologies, USA). The RNA isolated by both the methods was treated with DNase (Ambion^®^ Life Technologies, USA) to remove contaminating genomic DNA. cDNA was synthesized with the High capacity cDNA Reverse transcription kit from Applied Biosystems (Foster City, CA, USA). Real-time qPCR was performed by SYBR Green technology (Takyon Low ROX SYBR mix, Eurogentec) and Stratagene MxPro 3000 instrument (Agilent Technologies, USA) in cDNA from isolated samples. Primers used are given in S1 Table. The relative fold change in expression of gene of interest in samples with respect to calibrator was calculated using the formula 2^-ΔΔCt^.

### Microarray hybridization and data analysis

Microarray hybridization was conducted on arrays (AMADID 28005 & 26986) using Agilent Platform (Agilent Technologies, CA) and data was analyzed using GeneSpring GX software by Genotypics Pvt Ltd, Bangalore, India. The sample labeling was performed using Quick-Amp Labeling Kit, One Color (Agilent Technologies, CA, USA). cRNA was generated by *in vitro* transcription and the dye Cy3 CTP incorporated. The Cyanine 3-CTP labeled cRNA sample was purified using Qiagen RNeasy column (Qiagen, Limberg, Netherlands). For hybridization, labeled Cyanine 3-CTP cRNAs were fragmented to an average size of approximately 50 to 100 nucleotides hybridized onto array (AMADID 28005 & 26986). The hybridization was carried out at 65°C for 16 hours and the slides were washed and scanned using Agilent Scanner (Agilent Technologies). Data extraction from Images was done using Agilent Feature Extraction software Version 11.5.1.1. Images were quantified using Feature Extraction Software (Version-11.5, Agilent). Feature extracted raw data was analyzed using GeneSpring GX software from Agilent.

Normalization of the data was done in GeneSpring GX using the 75th percentile shift. Differential expression patterns were identified among the samples. Significant genes up regulated fold > 0.6 (logbase2) and down regulated <-0.6 (logbase2) in the test samples with respect to control sample were identified. ‘p’ value of statistical student t-test among the replicates was calculated based on volcano plot algorithm. Differentially regulated genes were clustered using hierarchical clustering based on Pearson coefficient correlation algorithm to identify significant gene expression patterns. Pathway analysis for the differentially regulated genes was performed using Genotypic Biointerpreter-Biological Analysis Software. The Significant functional classification of differentially regulated genes was performed using GeneSpring GX software gene ontology and Database for Annotation, Visualization and Integrated Discovery (DAVID) v6.7. R program was used to generate scatter plots of log2 transformed expression values. Raw data is deposited in GEO data base (accession ID: GSE73543).

### Chromatin Immuno-Precipitation (ChIP)

Cells were pelleted and resuspended in 500 μl of PBS containing 20 mM sodium butyrate. Cells were fixed at room temperature with 1% formaldehyde for 8 minutes, and then quenched with 1.25 M glycine. Cells were sonicated using Misonix sonicator under the following conditions to obtain chromatin fragments between 200–700 bp size: Hepatocytes– 65 Amplitude (4 pulses with 1 minute interval between each pulse which was of 16 seconds with 1 second on and 1 second off); Lin^-^ BM cells– 40 Amplitude (1 pulse of 10 minutes with 30 seconds on and 30 seconds off). ChIP was performed using Low cell ChIP kit from Diagenode (Seraing, Belgium) according to the manufacturer’s protocol using the specific antibodies. Antibodies used for ChIP (anti-JMJD3, anti-EZH2, anti-H3K4me3, anti-H3K9Ac, anti-H3K9me3, anti-H3K27me3, rabbit IgG) were procured from Abcam (Cambridge, UK). Analysis of ChIP samples was carried out by real-time qPCR using the primers (S2 Table) and the efficiency of chromatin immuno-precipitation was calculated as percentage of input (ChIP/Total input) = 2^[(Ct(1% input)–log_2_ (dilution factor))—Ct(ChIP)]^ x 100%.

### *In vitro* hepatic differentiation of Lin^-^ BM cells

Lin^-^ BM cells were cultured in the presence of Williams E medium (Gibco-BRL) containing 10% fetal bovine serum, 1× insulin-transferrin-selenium supplement (Invitrogen, CA), 20 ng/ml hepatocyte growth factor, 30 ng/ml oncostatin M, 10 ng/ml epidermal growth factor (R & D Systems, Minneapolis, Minnesota, USA), 10^-8^M dexamethasone, 10^-4^M ascorbic acid-2-phosphate (Sigma- Aldrich). Depending on the nature of the experiment, 100 nM GSK-J4 (Sigma-Aldrich) was added in the medium. Prior to culture, plates were coated with laminin-hyaluronic acid-collagen I.

### Periodic acid–Schiff staining for liver glycogen

Staining was performed using PAS reagent (Sigma Aldrich). Culture medium was removed and adhered cells were washed with PBS and air dried. Cells were then fixed with formalin-ethanol fixative for 1 minute at room temperature and rinsed with tap water. Periodic acid was added to cells and left for 5 minutes at room temperature. Cells were washed with distilled water, Schiffs reagent added and left for another 15 minutes at room temperature. Cells were washed and counterstained with hematoxylin for 90 seconds, washed with tap water, air dried and examined microscopically.

### Indocyanin Green (ICG) uptake and release

Indocyanin green dye (Sigma Aldrich) was added to the cells in culture at a concentration of 1 mg/ml and incubated at 37°C for 1 h. Medium with ICG was discarded and cells were washed with PBS and observed under microscope for cellular uptake of ICG. After that cells were further cultured in fresh medium 6 h at 37°C. The PBS-washed cells were observed again under microscope for release of the dye.

### Statistics

Results of multiple experiments were reported as mean ± SEM. Student’s t-test was carried out to calculate the significance between the means of both groups and p < 0.05 was considered as significant. All analyses were carried out using GraphPad Prism software, Version 5.02.

## Results

### Lin^-^ BM cells undergo partial reprogramming to form hepatocyte-like cells

**Fig 1:**
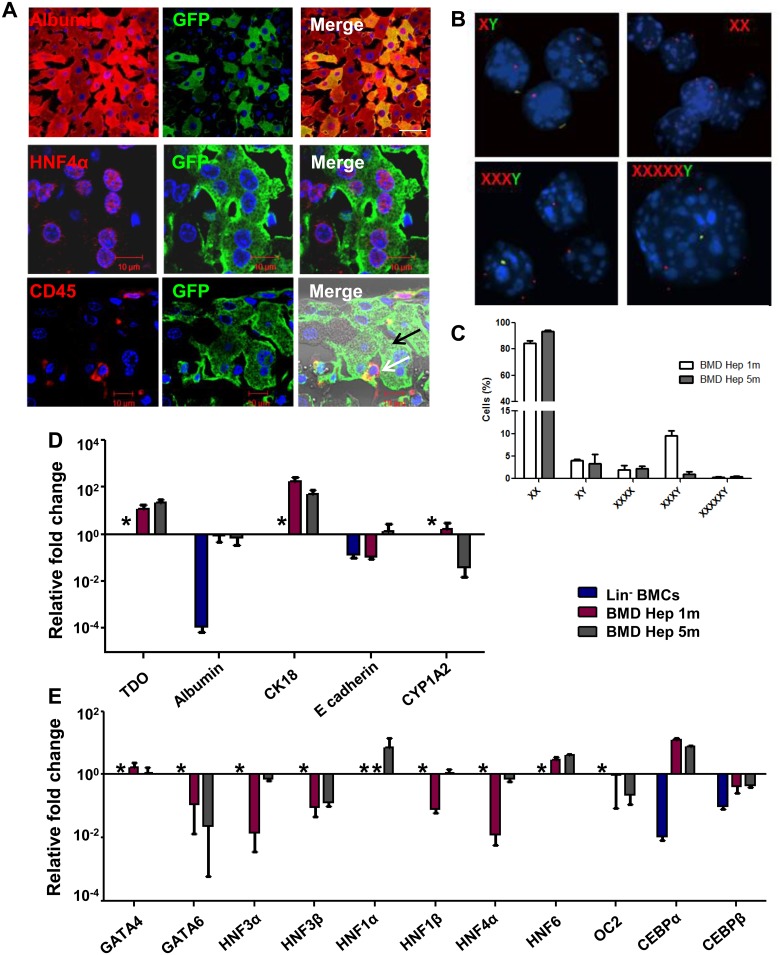
Generation of hepatocyte-like cells from Lin^-^ BM cells. (A) Immuno-histochemical analysis of recipient mice liver sections after transplantation. *Upper panel*: Images (600× magnification) showing donor derived hepatocytes (anti-GFP/donkey anti-mouse Alexa fluor 488 and anti-albumin /donkey anti-goat Alexa fluor 594). *Middle panel*: Images (630×2.8 zoom magnification) showing donor derived hepatocytes (anti-GFP/donkey anti-mouse Alexa fluor 488 and anti-HNF4α /donkey anti-goat Alexa fluor 594). *Lower panel*: IHC analysis of liver sections (630× magnification) from recipient showing donor derived hepatocytes which shows no CD45 expression (black arrow) and CD45 expressing non-parenchymal cells (white arrow). Number of experiment = 3. (B) Interphase FISH analysis of X and Y chromosome in BM-derived hepatocytes after 5 month of transplantation. The cells were analyzed for the presence of X (Red, platinum bright 550) and Y (Green, platinum bright 495) chromosomes in the nuclei (blue). (C) Cytogenetic analysis showing the percentage of donor cells that did not contain Y chromosome. Number of experiment = 3 (each experiment was based on pooled cells of 3 mice). (D) RT-qPCR analysis showing expressions of hepatic marker genes in BM-derived hepatocytes relative to expression in primary hepatocytes. Number of experiment = 3 (each experiment was based on pooled cells of 3 mice). (E) RT-qPCR analysis showing expression of various hepatic transcription factors in BM-derived hepatocytes relative to expression in primary hepatocytes. Number of experiment = 3 (each experiment was based on pooled cells of 3 mice). * No Ct.

After 5 months of transplantation (Tx) of eGFP-expressing Lin^-^ BMCs from female to hemophilia A (HA) male mice, the immuno-histochemical analysis of liver sections revealed the presence of donor-derived cells expressing hepatic markers, like albumin and HNF4α (Fig 1A, upper and middle panel). GFP^+^ cells that morphologically resembled hepatocytes did not express CD45, whereas few donor-derived non-parenchyma cells retained CD45 antigen expression (Fig 1A, lower panel). To ensure that the donor-derived cells lost mesenchymal phenotype, the liver sections were further stained for vimentin. The result confirmed that engrafted donor cells lost mesenchymal phenotype as they do not express vimentin protein (S1 Fig).

To investigate the chromosomal status of BM-derived hepatic cells, we performed fluorescent *in situ* hybridization (FISH) to identify XY chromosomes in sorted donor-derived cells (S2 Fig) after 1 and 5 months of Tx. Five month samples mostly possessed distinct nuclei with XX chromosomes (Fig 1B, *top right*), whereas few nuclei bearing multiple X chromosomes along with Y chromosome were also observed (Fig 1B, *bottom*). Cytogenetic analysis using 500 nuclei per mice revealed 10% of total donor-derived cells got fused with recipient cells that contained multiple X chromosomes along with Y chromosome. Whereas, remaining 90% of these were devoid of Y chromosome, suggesting that most of these donor-derived hepatic cells possessed female chromosome (Fig 1C). After 5 months of Tx, the fusion hybrids were reduced to about 3%. Thus, BM-derived hepatic cells after 5 months of transplantation were found to be highly homogeneous with respect to X chromosome content. We presume that these multiple X chromosomes bearing donor-derived hepatic cells were formed from the pathway of ploidy reduction during division of fusion hybrids between donor BM and host hepatic cells or by direct differentiation of BM cells or by combination of both in present experimental model.

To examine time dependent maturation of BM-derived hepatic cells, we conducted comparative gene expression analyses of few liver specific markers (*TDO*, *Albumin*, *CK18*, *E-cadherin and CYP1A2*) and major hepatic transcription factors (*GATA4*, *GATA6*, *HNF3α*, *HNF3β*, *HNF1α*, *HNF1β*, *HNF4α*, *HNF6*, *OC-2*) in Lin^-^ BM cells and BM-derived hepatic cells with respect to primary hepatocytes. The expressions of these genes were observed in BM-derived hepatocytes albeit to varying degrees (Fig 1D and 1E). These results suggested that an endodermal transcriptional switch reprogrammed Lin^-^ BM cells to hepatocyte-like cells.

### Genome-wide transcriptional remodeling occurs in BM-derived hepatocytes within one month of transplantation

Whole genome microarray analysis was performed to characterize the global transcriptional profile of BM-derived hepatocytes. The Lin^-^ BM cells clustered separately from the primary hepatocytes and BM-derived hepatocytes, whereas later two clustered together indicating that BM-derived hepatocytes were closer to primary hepatocytes in gene expression profile (S3A Fig). Differential gene clustering also revealed the dynamic trend in expression profiles of various gene clusters during mesodermal to hepatic lineage conversion (S3A Fig). Global gene analyses also revealed that differential gene expression was observed in BM-derived hepatocytes post 1 month of Tx, which was also noticed at a later time point suggesting that lineage conversion occurred very early under permissive micro-environment cues (S3B Fig). Gene ontology analysis showed that the up-regulated genes in BM-derived hepatocytes belonging to major metabolic process (*Aldh8a1*, *Sc5d*, *Hmgcr*, *Cyp2d9*, etc.) and acute inflammatory response (*CRP*, *C9*, etc.) (S4A and S4B Fig). The commonly up-regulated genes in BM-derived hepatocytes after 1 and 5 months of Tx are mainly involved in lipid and steroid metabolism, like *Acadm* and *Hmgcs1* (S4C Fig). The major down-regulated genes in BM-derived hepatocytes were involved in leukocyte activation (*Stat5α*, *Jag2*, etc.), cell cycle and cell division (*Cdk6*, *E2F4*, etc.), transcriptional regulation (*Creb1*, *Tcf4*, etc.) and chromatin organization (*Cbx4*, *Ino80*, etc.) as shown in S5A and S5B Fig. The commonly down-regulated genes in BM-derived hepatocytes after 1 and 5 months of transplantation were found to be involved in DNA and RNA synthesis, processing (*Cbx4*, *RNASEL*, etc.) and chromosome reorganization (*Arid1α*, *Cbx7*, etc.) (S5C Fig).

**Fig 2:**
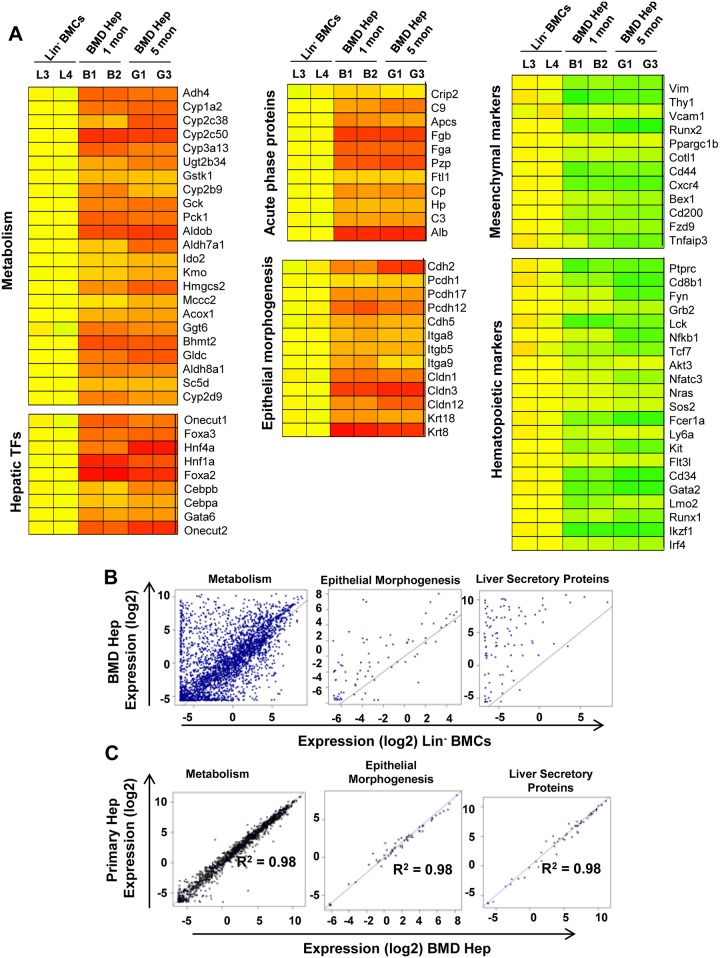
Genome-wide transcriptional remodeling in BM-derived hepatocytes relative to Lin^-^ BM cells. (A) Heat map of differentially regulated hepatocyte-specific functional genes and mesenchymal and hematopoietic lineage specific genes in BM derived hepatocytes– 1 month (B1, B2) and 5 month (G1, G3) with respect to control–Lin^-^ BMCs (L3, L4). (B) Scatter plot analysis generated by R program comparing the log2 transformed normalized expression values of the filtered genes in Lin^-^ BMCs and BM-derived hepatocytes (1 month). (C) Scatter plot analysis generated by ‘R’ program comparing the log2 transformed normalized expression values of the filtered genes in primary and BM-derived hepatocytes (5 month). Number of experiment = 2 (each experiment was based on pooled cells of 3 mice).

Transcriptional profiling showed that hepatic TFs and functional genes involved in metabolism, epithelial morphogenesis and secretory functions were upregulated in BM derived hepatocytes, whereas hematopoietic and mesenchymal genes were down-regulated relative to that in Lin^-^ cells (Fig 2A). On further analysis of genes with functional significance in hepatocytes by ‘R’ program, an induction of expression of genes involving metabolism, epithelial morphogenesis and secretory proteins were seen in BM-derived hepatic cells when compared to Lin^-^ BM cells within 1 month of Tx (Fig 2B). Interestingly, analysis by ‘R’ program also showed a high degree of similarity in expression of above functional genes in 5 months samples of BM-derived hepatocytes and primary hepatocytes (Fig 2C). Subsequent analysis revealed that BM-derived hepatocytes retained expression of certain genes that have functional significance in hematopoietic system like cytokines receptors and chemokine signaling pathways (S6A and S6B Fig). These results suggest that BM-derived cells progressively matured to primary hepatocyte-like cells.

The real time PCR results validated microarray data and ensured similarity in major functional gene expressions among BM-derived and primary hepatocytes (S6C and S6D Fig). It is important to note that microarray results revealed down-regulation of hematopoietic genes (*CD45* and *GATA2*) in BM-derived hepatic cells.

### Dynamic epigenetic changes occur at the promoters of HTFs

**Fig 3:**
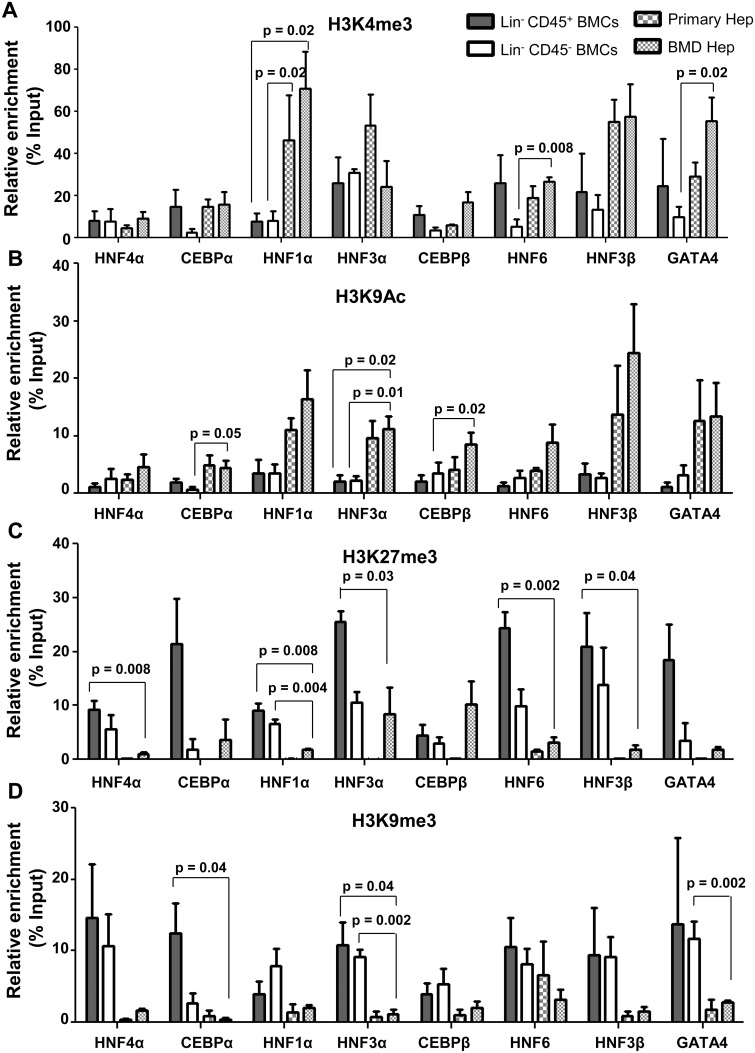
Histone modifications at promoters of hepatic transcription factors in BM-derived hepatocytes. BM-derived hepatocytes were isolated after 5 months of transplantation for ChIP-qPCR analysis. Lin^-^CD45^+^ and Lin^-^CD45^-^ BMCs served as negative controls and primary hepatocytes as positive control. ChIP-qPCR analysis of (A) H3K4me3, (B) H3K9Ac, (C) H3K27me3 and (D) H3K9me3 at the promoters of hepatic transcription factors in BM-derived hepatocytes. Enrichment of the marks in the immuno-precipitated samples over input samples was calculated. Number of experiment = 3 (each experiment was based on pooled cells of 3 mice). Refer to S3 Table for IgG control values.

As differentiation of cells is a consequence of epigenetic changes, we examined whether genetic changes that have occurred in BM-derived hepatocytes is also supported at epigenetic level. We analyzed the enrichment of activating histone [H3K4me3 and H3K9Ac] and repressive histone [H3K9me3 and H3K27me3] marks at the promoters of developmentally important hepatic transcription factors (HTFs) like *HNF4α*, *CEBPα*, *HNF1α*, *HNF3α*, *CEBPβ*, *HNF6*, *HNF3β* and *GATA4* in BM-derived hepatocytes after 5 months of Tx. As Lin^-^ BM cells used for transplantation have both Lin^-^CD45^+^ (hematopoietic) and Lin^-^CD45^-^ (mesenchymal) fractions, we isolated these two fractions (S7A Fig) and transplanted them separately in HA mice. After one month of transplantation it was observed that both cell fractions were able to engraft and generate hepatocyte-like cells (S7B Fig). Since both fractions of Lin^-^ cells gave rise to hepatocytes, we separately conducted epigenetic characterization of Lin^-^CD45^+^ and Lin^-^CD45^-^ cell fractions as controls since they had very different epigenetic landscapes. Donor Lin^-^ BM cell-derived hepatocytes were used as the test samples for this analysis, which were expected to have similar reprogramming marks at hepatic gene promoters after differentiation of both fractions of cells. ChIP-qPCR analysis showed that the levels of H3K4me3 at HTF gene promoters in Lin^-^CD45^+^ cells were similar to that in BM-derived hepatocytes except for HNF1α. However, Lin^-^CD45^-^ cells had lower levels of H3K4me3 to that in BM-derived hepatocytes though significant differences were observed only at the promoters of HNF1α, HNF6 and GATA4 (Fig 3A). H3K9Ac was significantly enriched at the HTF promoters in primary hepatocytes as well as in BM-derived hepatocytes, whereas only a minimal level of enrichment was observed in Lin^-^CD45^+^ and Lin^-^CD45^-^ BM cells (Fig 3B).

ChIP-qPCR analysis also showed that the silencing histone mark H3K27me3 was significantly enriched (5 to 25% of input) at the promoters of all major hepatic transcription factors in Lin^-^CD45^+^ and Lin^-^CD45^-^ cells. In primary hepatocytes only a minimal enrichment of H3K27me3 mark was observed at these promoters, whereas in BM-derived hepatocytes H3K27me3 mark was still present at the promoters of *CEBPα*, *HNF3α* and *CEBPβ*. Other promoters like *HNF4α*, *HNF1α*, *HNF6*, *HNF3β* and *GATA4* showed only minimal levels of H3K27me3 enrichment (Fig 3C). H3K9me3 was highly enriched (5 to 15% of input) at all the hepatic transcription factor gene promoters in both Lin^-^CD45^+^ and Lin^-^CD45^-^ cells, but only minimally enriched at these promoters in primary hepatocytes and BM-derived hepatocytes (Fig 3D).

As real time PCR results confirmed silencing of *CD45* and down-regulation of *GATA2* genes in BM-derived hepatocytes, we determined the change of histone mark occupancy at the promoter levels. No significant change in the enrichment levels of H3K4me3 and H3K9Ac marks were noticed, whereas H3K27me3 (8 and 53% of input) and H3K9me3 (15% and 25% of input) levels were significantly enriched at promoters of *CD45* and *GATA2* in BM-derived hepatocytes compared to Lin^-^CD45^+^ cells (S8 Fig). This suggests that expression of *CD45* and *GATA2* was repressed by high enrichment levels of silencing histone marks. The anti-rabbit IgG control data indicated a minimal enrichment of histone marks over input samples (S3 Table).

Interestingly, ChIP-qPCR analysis revealed the presence of a bivalent mark for poised gene expression, which is the presence of the activating mark H3K4me3 and the repressive mark H3K27me3 at the HTF promoters in hematopoietic progenitor cells (Fig 3A and 3C). This suggests that these genes were in an epigenetically poised state for expression. Removal of H3K27me3 and H3K9me3 from these promoters resulted in transcriptional activation and expression of liver enrichment transcription factors.

### Role of chromatin modifying enzymes in induction of epigenetic reprogramming

**Fig 4:**
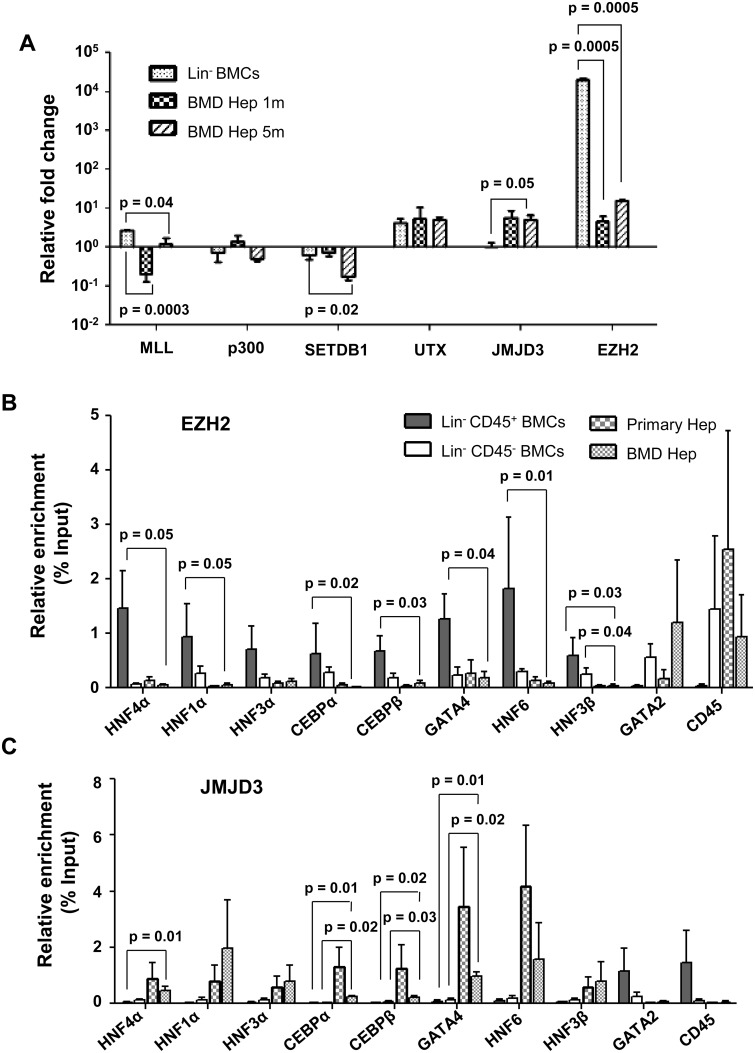
Chromatin modifying enzymes in reprogramming of Lin^-^ BMCs to hepatocytes. (A) Expression of some chromatin modifying enzymes in BM-derived hepatocytes. Expression levels normalized to that of primary hepatocytes were used to calculate relative fold change. (B) ChIP-qPCR analyses of EZH2 at the promoters of hepatic transcription factors and hematopoietic genes in BM-derived hepatocytes. Enrichment of the marks in the immuno-precipitated samples over input samples was calculated. (C) ChIP-qPCR analyses of JMJD3 at the promoters of hepatic transcription factors and hematopoietic genes in BM-derived hepatocytes. Number of experiment = 3 (each experiment was based on pooled cells of 3 mice). Refer to S4 Table for IgG control values.

Potential chromatin modifying enzymes that could establish the histone code at selected gene promoters during cellular reprogramming of BM cells to hepatocyte-like cells were analyzed. Out of 6 such enzymes examined, the expressions of *MLL*, *p300*, *SetDB1* and *UTX* were either down-regulated or unchanged in BM-derived hepatocytes when compared with Lin^-^ BM cells (Fig 4A). Interestingly, 8 folds up-regulation of *JMJD3 (KDM6B)*, an H3K27me3 demethylase, and 1000-fold reduction in the expression of *EZH2*, an H3K27 methyltransferase was found in BM-derived hepatocytes compared to Lin^-^ BM cells (Fig 4A). The gene expression analyses of chromatin modifying enzymes (JMJD3 and EZH2) along with the promoter analysis for histone occupancy (Fig 3C) suggested that the loss of H3K27me3 (silencing mark) from hepatic TF promoters was one of the important epigenetic driving mechanisms for cellular reprogramming.

In order to confirm the role of EZH2 and JMJD3 in lineage switch, ChIP-qPCR analysis was conducted at the promoters of *HNF4α*, *CEBPα*, *HNF1α*, *HNF3α*, *CEBPβ*, *HNF6*, *HNF3β* and *GATA4* to determine the binding of these proteins to the promoters. EZH2 was found to be enriched at these promoters in Lin^-^CD45^+^ cells, but in primary hepatocytes and BM-derived hepatocytes the enrichment for EZH2 was minimal at these loci (Fig 4B). It was also found that EZH2 was bound to *CD45* and *GATA2* promoters in Lin^-^CD45^-^ cells, primary hepatocytes and BM-derived hepatocytes (Fig 4B).

Conversely, JMJD3 was enriched at HTF promoters in primary and BM-derived hepatocytes, but not in Lin^-^CD45^+^ and Lin^-^CD45^-^ cells (Fig 4C). Further analysis revealed the binding of JMJD3 to *CD45* and *GATA2* promoters in Lin^-^CD45^+^ cells, but not in primary hepatocytes or BM-derived hepatocytes (Fig 4C). The anti-rabbit IgG control data indicated only a minimal enrichment of histone marks over input samples (S4 Table). Above results led to an interesting hypothesis that binding of JMJD3 to HTF promoters is required for removal of silencing H3K27me3 mark that was established by EZH2 to facilitate epigenetic reprogramming of BM cells.

As expression of JMJD3 was relatively low in both Lin^-^ BM cells and primary hepatocytes when compared with BM derived hepatocytes, we anticipated its’ induction for epigenetic reprogramming during regeneration of liver after the injury. In fact liver regeneration experiment confirmed 1.5–2.2 fold increase of *JMJD3* gene expression as compared to normal liver (S9A Fig). This was supported by the induction of JMJD3 expression and subsequently nuclear localization along with HNF4α (S9B Fig). Other than regeneration, JMJD3 was found to be expressed more during maturation of liver (~18 dpc) as compared to 13 dpc fetal liver (S10A Fig). This was further confirmed by high nuclear expression of JMJD3 in the HNF4α-expressing cells in 18 dpc fetal liver (S10B Fig). On the contrary, EZH2 was high in 13 dpc fetal liver, which reduced in 18 dpc fetal liver (S10C Fig).

### JMJD3 is required for epigenetic reprogramming of BM progenitor cells to hepatocytes

The role of JMJD3 and its mechanism of action in epigenetic reprogramming of Lin^-^ BM cells were further examined by *in vitro* differentiation experiments in the presence GSK-J4, a pro-drug of GSK-J1 that inhibits catalytic H3K27me3 demethylase activity of JMJD3. Fourteen days culture of Lin^-^ BM cells in hepatic differentiation medium resulted in expression of hepatic genes and proteins (S11A and S11B Fig). Quantitative analyses revealed that 8% of the cells expressed HNF4α, 12% expressed albumin and 11% expressed E-cadherin after 14 days of culture (S11C Fig). Low level of hepatic differentiation in these cells suggesting that propensity for changing cellular phenotype *in vitro* was low. Functional assays established that at least a few differentiated cells were capable of performing hepatic functions such as storing glycogen, uptake and release of indocyanin green dye after 30 days of culture (S11D Fig).

**Fig 5:**
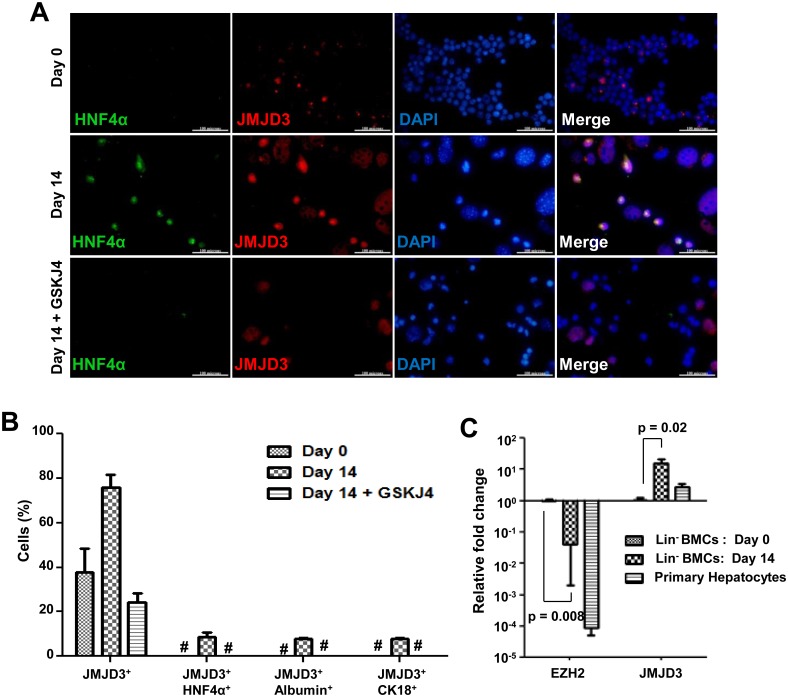
Role of JMJD3 during *in vitro* hepatic differentiation of Lin^-^ BMCs. (A) Expression of hepatic marker HNF4α and induction of JMJD3 as analyzed by immuno-cytochemistry after 14 days of culture with or without GSKJ4 inhibitor (scale = 100 μm, 600× magnification). Larger cells are hypertrophic and ignored from analysis. (B) Quantification of *in vitro* differentiated cells that express JMJD3 alone, and co-expressed HNF4α /JMJD3, albumin/JMJD3, CK18/JMJD3. Refer S8A and S8B Fig. *No cells co-expressed HNF4α and JMJD3. (C) Expression of the chromatin modifying enzymes, JMJD3 and EZH2 that regulate H3K27 methylation. Expression levels relative to Lin^-^ BMCs (day 0) are shown. Number of experiment = 3.

**Fig 6:**
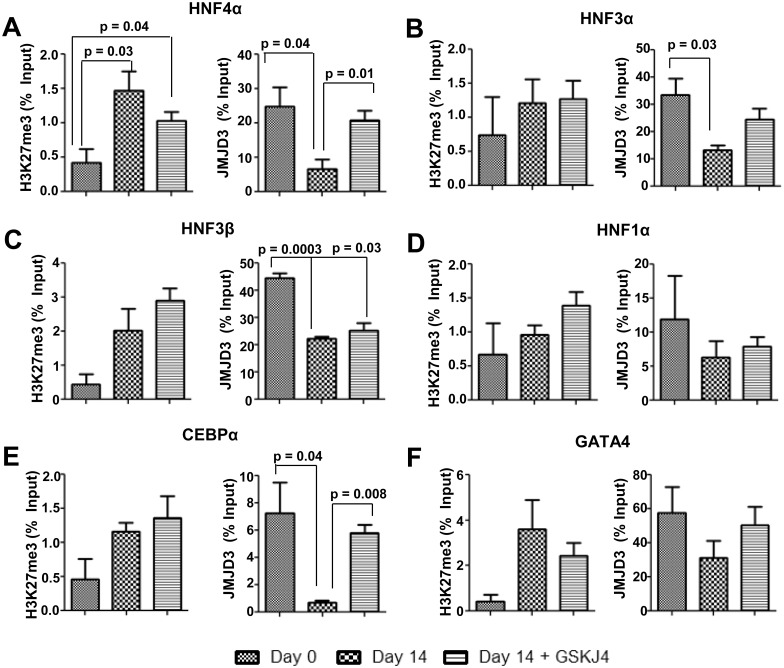
Binding of JMJD3 to HTFs in the presence of GSKJ4 inhibitor. Relative enrichment of H3K27me3 and JMJD3 at promoters of (A) HNF4α (B) HNF3α (C) HNF3β (D) HNF1α(E) CEBPα and (F) GATA4 determined by ChIP-qPCR. Number of experiment = 3. p value < 0.05 was considered as significant change. Refer to S5 Table for IgG control values.

Immuno-cytochemical analysis of 14 days cultured cells revealed an increase of the percentage of cells that expressed JMJD3, whereas in the presence of inhibitor GSK-J4, JMJD3 expression was inhibited along with the hepatic differentiation (Fig 5A and 5B, S12A and S12B Fig). A 15-fold increase in expression of JMJD3 with a concomitant down-regulation of EZH2 was also observed during reprogramming of Lin^-^ BM cells (Fig 5C). Though earlier reports have attributed the role of JMJD3 in differentiation by acting as a H3K27me3 demethylase, it has also been shown that JMJD3 can promote transcription, independent of its H3K27me3 demethylase activity [23]. To elucidate the mechanism involved in cellular reprogramming in the present system, we determined the binding of JMJD3 to various hepatic transcription factor promoters and analyzed its effect on H3K27me3 level by ChIP-qPCR in the presence and absence of GSK-J4. After 14 days of induction there was a significant activation of JMJD3 and increased binding of JMJD3 to these promoters with a concomitant loss of H3K27me3 from these loci. In the presence of JMJD3 inhibitor, though it is continued to bind to these promoters, H3K27me3 enrichments maintained at levels comparable to Lin^-^ BMCs (Fig 6A–6F). The IgG control data (S5 Table) showed only minimal enrichment. The loss of H3K27me3 chromatin mark from cells in the presence of inductive conditions was inhibited by GSK-J4, as shown by immuno-cytochemical analysis (S12C Fig). Overall, these results indicate that JMJD3 is needed for hepatic differentiation of Lin^-^ BM cells by active demethylation of H3K27me3 at promoters of HTFs.

## Discussion

In liver regeneration model, the acquisition of hepatic phenotype by BM-derived cells may take place following two pathways: (a) heterotypic fusion that lead to nuclear reprogramming towards hepatic lineage and (b) liver regeneration cues which trigger nuclear reprogramming in favor of hepatic differentiation. Liver regeneration by BM-derived cells has been documented for many years [2, 6, 9, 10–12, 15]; but little is known about the molecular mechanism that induces this fate change. The only report published so far in this regard had shown that heterotypic fusion-induced nuclear reprogramming led to the formation of cells, distinct from the primary hepatocytes [21].

Here we have studied the reprogramming of Lin^-^ BMCs for longer duration than earlier [21], so that the hepatic cells so formed can be compared with primary hepatocytes in term of genomic and epigenomic states. Global transcriptional analyses clearly indicate that though 1 month post-transplanted BM-derived hepatic cells expressed many hepatocyte-specific genes, nuclear reprogramming in these cells was not complete as many hematopoietic genes were still expressed. This could be due to heterogeneity of cells as a substantial fraction of them were fusion hybrids in which complete lineage change did not occur. The hepatocyte-like cells derived from embryonic stem cells (ESCs) or iPSCs or germ line cell-derived pluripotent stem cells or even fibroblasts were found to be comparable to the primary hepatocytes in expression of specific liver genes [4, 5, 24–27], as observed in this study using BM-derived cells. In injured liver milieu, different hepatotropic factors are believed to be involved in such phenotype conversion from BM-derived cells to hepatocytes as well as their maturation.

The comparative analyses of human hepatocyte-like cells, derived from ESCs or iPSCs, have revealed that despite significant similarities in gene expressions, there were considerable differences in the expressions of cytochrome P450 enzymes as compare to primary hepatocytes [26]. In another recent study, microarray analyses of BM-derived hepatocytes showed that over-expressed genes belonging to neuro-transmission, TGFβ signaling pathway and metalloproteases, whereas the down-regulated genes involve in chromatin organization, RNA processing and mitochondrial functions. Despite these differences in the donor-derived cells as compared to primary hepatocytes, hepatic repair function was attributed to them [21]. In contrast to above findings, the present study showed that after 5 months of Tx the BM originated hepatocyte-like cells were comparable to primary hepatocytes in terms of expression of genes related to metabolism, epithelial morphogenesis and liver secretory proteins. Though expression of a few hematopoietic receptor genes continued expression, their down-stream effects/adapter molecules were absent. Lineage reprogramming was also followed by down-regulation or silencing of donor (Lin^-^CD45^+^) signature genes like *GATA2* and *CD45*, which was also evidenced in induced hepatocytes obtained from fibroblasts [28] and in human ESC-derived hepatocytes [29].

Chromatin modifications have been shown to constitute an additional layer of transcriptional regulation. In this study, analysis of enrichment of various histone marks at the promoter regions of developmentally important hepatic transcription factors showed that permissive histone marks (H3K4me3 and H3K9Ac) were enriched at these loci whereas repressive marks (H3K27me3 and H3K9me3) were only minimally present at these sites in primary hepatocytes and BM-derived hepatocytes when compared to the Lin^-^ fraction of BM cells. These results were in support of the expression of HTFs in BM-derived hepatocytes. The findings also substantiate the role of chromatin modifications in driving differentiation or nuclear programming as clearly seen in several other systems [30–32]. Another interesting finding from this experiment was that H3K4me3 mark was enriched in Lin^-^CD45^+^ BM cells at most of the HTF promoters, which were also associated with H3K27me3 mark. It is known that bivalent mark is the presence of activating H3K4me3 and repressive H3K27me3 mark at the promoter that keeps the gene poised for differentiation under inductive conditions [33]. The presence of this bivalent mark suggests that though these genes were not actively expressed, they remained in a poised state of expression in Lin^-^CD45^+^ BM cells. Favorable conditions that facilitate the loss of H3K27me3 mark from these sites can lead to the activation of their transcription and promote reprogramming of BM progenitors to hepatocytes. Increase of silencing marks (H3K27me3 and H3K9me3) at hematopoietic gene promoters in BM-derived hepatocytes implied that epigenetic changes during nuclear reprogramming also induced repressive alterations at donor signature gene promoters.

Among all the chromatin remodeling enzymes analyzed, an interesting inverse co-relation was observed between JMJD3 and EZH2 that regulate methylation status of H3K27. The up-regulation of JMJD3 and down-regulation of EZH2 suggest an important role for JMJD3 in removal of H3K27me3 marks from promoters of cells that undergo reprogramming. This hypothesis was supported by ChIP-qPCR results, which showed increase in binding of JMJD3 to the HTF promoters in both primary and BM-derived hepatocytes whereas these loci were occupied by EZH2 in the Lin^-^CD45^+^ and Lin^-^CD45^-^ BM cells. EZH2 facilitated establishing of H3K27me3 mark at these sites through its H3K27 methyltransferase activity in Lin^-^ BM cells. Down-regulation of EZH2 in hepatic progenitor cells has been reported to induce differentiation and maturation into functionally active hepatocytes [34]. On the other hand, JMJD3 was shown to have a critical role in induction of definitive endoderm from ESCs [35] and also in the development of pancreas [36] and lungs [37], which are the organs of endoderm origin. Until date no report has been published indicating the significance of JMJD3 in hepatic differentiation and liver development. For the first time, this study shows the presence of JMJD3 in developing fetal liver and recognizes a probable role of JMJD3 in converting the cellular framework of fetal liver from hematopoietic to epithelial constitution. As usual, JMJD3 was enriched in case of hematopoietic genes *GATA2* and *CD45* promoters in Lin^-^CD45^+^ BM cells, however during reprogramming recruitment of EZH2 led to establishment of H3K27me3 mark at these sites to down-regulate/silence their expression.

Induction of epigenetic reprogramming may occur both in fusion-dependent and fusion-independent pathways follow for the formation of hepatocytes from Lin^-^ BM cells *in vivo*. A few reports have suggested that cell fusion can lead to changes in DNA methylation patterns, which instruct nuclear reprogramming in heterokaryons leading to lineage conversion [38, 39]. Since DNA methylation patterns, the stable epigenetic marks, can be reprogrammed due to fusion, it is presumed that flexible marks like histone modifications may also undergo dynamic transitions during reprogramming. This study clearly showed an induction as well as nuclear localization of JMJD3 in hepatocytes during acute liver injury. Those hepatocytes formed due to fusion of BM cells and host hepatocytes, the induced JMJD3 was believed to catalyze the removal of H3K27me3 marks from the HTF promoters in former cell type, thus instructing them to attain the hepatic phenotype. As JMJD3 is a crucial enzyme that can remove the silencing mark H3K27me3, its induction can have direct implications on gene expression during hepatic differentiation of BMCs. *In vitro* hepatic differentiation experiments of Lin^-^ cells clearly demonstrated the propensity of a smaller fraction of BMCs to differentiate into functionally active hepatocyte-like cells. ChIP experiments in the presence of JMJD3 inhibitor has confirmed that the mechanism by which JMJD3 promoted hepatic differentiation was through active demethylation of H3K27me3 from HTF promoters rather than functioning as a transcription factor, though GSKJ4 may have certain non-target inhibitory effects that was not analyzed in this study. Further elaborate studies are needed by generating conditional JMJD3 knockout mouse model to dissect the stage-specific mechanisms involved in fetal liver development and BM-specific deletion of JMJD3 prior to transplantation will validate the *in vivo* functional role during liver regeneration. Another limitation of this study was that we have not separately characterized the hepatocytes obtained from Lin^-^CD45^+^ and Lin^-^CD45^-^ transplanted groups, which needs to be pursued in the future. Furthermore, elaborate and systematic characterization is needed to determine the differences in the lineage conversion mechanism and factors involved when these two cell fractions are separately transplanted.

## Conclusions

The comprehensive gene expression analysis revealed the dynamic transcriptional changes in BM-derived hepatocytes and their similarity with the primary hepatocytes after reprogramming. Targeted epigenetic analysis of some important histone modifications at gene promoters of major HTFs provided valuable insights in the changes of their chromatin landscape and the role of chromatin modifying enzymes in induction of cellular reprogramming. For the first time, this study implicates the potential role of JMJD3 in epigenetic reprogramming during lineage conversion of Lin^-^ BMCs to hepatocytes in regenerating liver.

## Supporting information

S1 Fig Expression of vimentin by donor-derived cells. Mice were transplanted with Lin^-^ BM cells. After 5 months of transplantation the liver sections were stained with eGFP and vimentin specific antibodies.
(TIF)

S2 Fig Isolation of BM-derived hepatocytes. (A) Single cell suspension of hepatocytes was made by two-step collegenase perfusion and the donor BM-derived hepatocytes were isolated by flow cytometry on the basis of eGFP marker. (B) The sorted cells were stained for GFP and albumin markers (anti-GFP/donkey anti-mouse Alexa fluor 488 and anti-albumin /donkey anti-goat Alexa fluor 594) to further analyze the purity of post sort samples.
(TIF)

S3 Fig Genome-wide transcriptional remodeling in BM-derived hepatocytes relative to Lin^-^ BM cells. (A) Hierarchical clustering of differentially regulated genes in BM derived hepatocytes– 1 month (B1, B2) and 5 month (G1, G3), primary hepatocytes (H2, H4) with respect to control–Lin^-^ BMCs (L3, L4). (B) Venn diagram illustrating the overlap in differential gene expression profile between BM–derived hepatocytes after 1 month and 5 months of transplantation relative to Lin^-^ BMCs.
(TIF)

S4 Fig Functional annotation of up-regulated genes in BM derived hepatocytes. Gene ontology analysis of the total up-regulated genes in BM derived hepatocytes after (A) 1 month and (B) 5 months of transplantation with respect to Lin^-^ BM cells. (C) Gene ontology analysis of the commonly up-regulated genes in BM derived hepatocytes after 1 and 5 months of transplantation with respect to Lin^-^ BM cells. Number of experiment (n) = 2.
(TIF)

S5 Fig Functional annotation of down-regulated genes in BM derived hepatocytes. Gene ontology analysis of the total down-regulated genes in BM derived hepatocytes after (A) 1 month and (B) 5 months of transplantation with respect to Lin^-^ BM cells. (C) Gene ontology analysis of the commonly down-regulated genes in BM derived hepatocytes after 1 and 5 months of transplantation with respect to Lin^-^ BM cells. Number of experiment (n) = 2.
(TIF)

S6 Fig Differential expressions of genes in BM-derived hepatocytes and qPCR validation of microarray results. (A) Functional annotation of these genes obtained by DAVID Bioinformatics Resources 6.7. Number of experiment (n) = 2. (B) Heat map of hematopoietic genes, the expression of which is retained in donor derived hepatocytes even after 5 months of transplantation. Number of experiment (n) = 2. (C & D) Fold change in expression of few specific hepatic genes in BM-derived hepatocytes relative to Lin^-^ BM cells after 1 and 5 months of transplantation. Number of experiment (n) = 3.
(TIF)

S7 Fig Engraftment of CD45^+^ and CD45^-^ fractions of Lin^-^ BMCs in damaged liver. **(A)** Flow cytometric analysis for CD45^+^ and CD45^-^ fractions of cells present in Lin^-^ BMCs. (B) Engraftment of Lin^-^CD45^+^ and Lin^-^CD45^-^ fractions of Lin^-^ cells in damaged liver of mice after 1 month of transplantation and albumin expression by the engrafted cells. Number of mice per group = 3.
(TIF)

S8 Fig Histone modifications at promoters of hematopoietic genes in BM-derived hepatocytes. BM-derived hepatocytes are isolated after 5 months of transplantation for ChIP-qPCR analysis. Lin^-^CD45^+^ and Lin^-^CD45^-^ BM cells served as negative controls and primary hepatocytes as positive control. ChIP-qPCR analyses of (A) H3K4me3 (B) H3K9Ac (C) H3K27me3 and (D) H3K9me3 at the promoters of hematopoietic genes in BM-derived hepatocytes. Enrichment of the marks in the immuno-precipitated samples over input samples has been calculated. Number of experiment (n) = 3. S1 Table for IgG controls.
(TIF)

S9 Fig Involvement of JMJD3 in hepatic differentiation during development and regeneration. (A) Expression of *JMJD3* in livers harvested day 1 and day 4 post induction of injury by acetaminophen relative to that of primary hepatocytes. (B) Immuno-histochemical analysis of liver cryo-sections to study expression of JMJD3 in hepatocytes after liver injury (scale = 100μm, 600X magnification). p value < 0.05 was considered as significant change. Number of animals used for analysis in each group = 5. Data are represented as mean ± SEM.
(TIF)

S10 Fig JMJD3 in fetal liver development. (A) Expression of JMJD3 in fetal livers of 13 and 18dpc mouse embryos relative to expression in normal adult liver. (B) Expression of EZH2 in fetal livers of 13 and 18dpc mouse embryos relative to expression in normal adult liver. (C) Immuno-histochemical analysis of 18dpc fetal liver cryo-sections to study expression of JMJD3 during liver development (scale = 100μm, 600× magnification). Number of mice = 3.
(TIF)

S11 Fig *In vitro* hepatic differentiation of Lin- BMCs. (A) Lin- BMCs were isolated and cultured on plates coated with hyaluronic acid, laminin and collagen I under hepatic differentiation conditions. After 14 days of culture cells were harvested and RNA was isolated. Expression of hepatic markers like HNF4α, HNF3α, albumin, E cadherin and CK18 was determined. Expression levels were calculated relative to Lin- BMCs. *denotes that no Ct was observed in these samples. Number of experiments = 3. (B) Expression of hepatic markers like HNF4α, albumin and E cadherin after 14 days of culture was determined by immuno-cytochemistry (scale = 100μm, 600X magnification). (C) Quantification of *in vitro* differentiated cells was performed using Image J 1.48 version. Number of experiments = 3. (D) Functional characterization of hepatocytes derived from Lin^-^ BMCs. Periodic acid schiff staining was performed in control Lin^-^ BMCs, test samples in which Lin^-^ BMCs were cultured under hepatic differentiation conditions for 30 days and primary hepatocytes (upper panel) (Scale = 100μm; 600X magnification). Indocyanin green uptake was assessed in these cells after 30 days of culture in control and test with primary hepatocytes as positive control (lower panel) (Scale = 200μm; 600X magnification). Number of experiments = 3. Data are represented as mean ± SEM.
(TIF)

S12 Fig Role of JMJD3 in hepatic differentiation of Lin- BMCs. Expression of hepatic markers (A) albumin and (B) CK18 and induction of JMJD3 as analyzed by immuno-cytochemistry in cyto-spun cells after 14 days of culture (scale = 100μm, 600× magnification) in presence and absence of GSKJ4. (C) Change in nuclear levels of H3K27me3 after 14 days of culture of Lin- BMCs under hepatic differentiation conditions with and without GSKJ4 was determined by immuno-cytochemical analysis (Scale = 100μm, 600X magnification). Cyto-spun cells were stained with rabbit anti-H3K27me3/anti-rabbit Alexa Fluor 594. Number of experiments = 3.
(TIF)

S1 Table Primers for analysis of gene expression by real time PCR. (DOC)

S2 Table Primers for analysis promoters of ChIP DNA. (DOC)

S3 Table Percentage enrichment of input in rabbit IgG controls for ChIP-qPCR analysis of histone marks. (DOC)

S4 Table Percentage enrichment of input in rabbit IgG controls for ChIP-qPCR analysis of binding of EZH2 and JMJD3 to gene promoters. (DOC)

S5 Table Percentage enrichment of input in rabbit IgG controls for ChIP-qPCR analysis of binding of JMJD3 and H3K27me3 to gene promoters. (DOCX)
