# Supplementary figures and images for: JMJD3 aids in reprogramming of bone marrow progenitor cells to hepatic phenotype through epigenetic activation of hepatic transcription factors
Source: PLoS One. 2017 Mar 22;12(3):e0173977. doi: 10.1371/journal.pone.0173977 (PMC5362104; doi:10.1371/journal.pone.0173977)

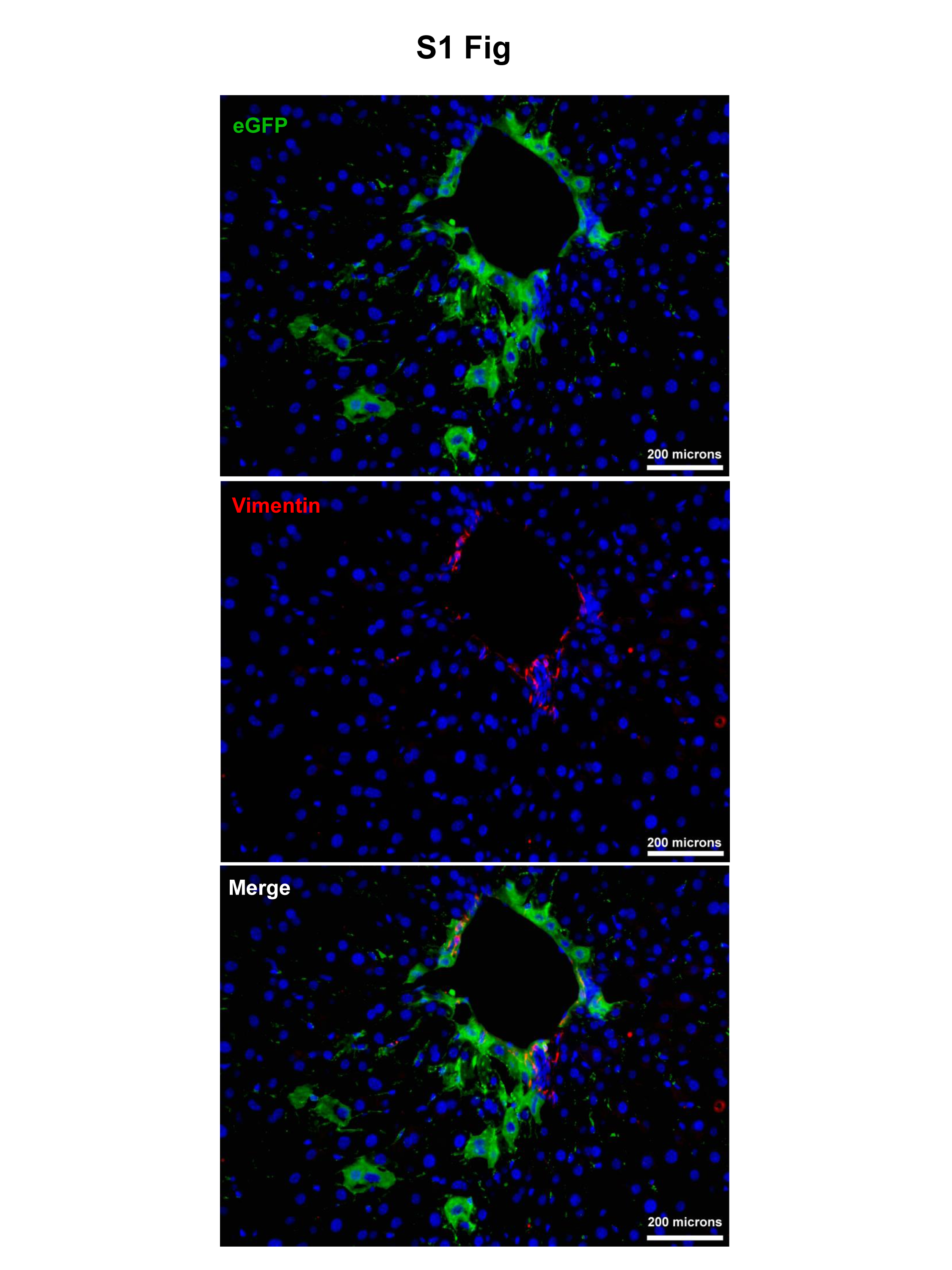

Supplement: S1 Fig — Mice were transplanted with Lin- BM cells. After 5 months of transplantation the liver sections were stained with eGFP and vimentin specific antibodies. (TIF) [file pone.0173977.s001.tif]

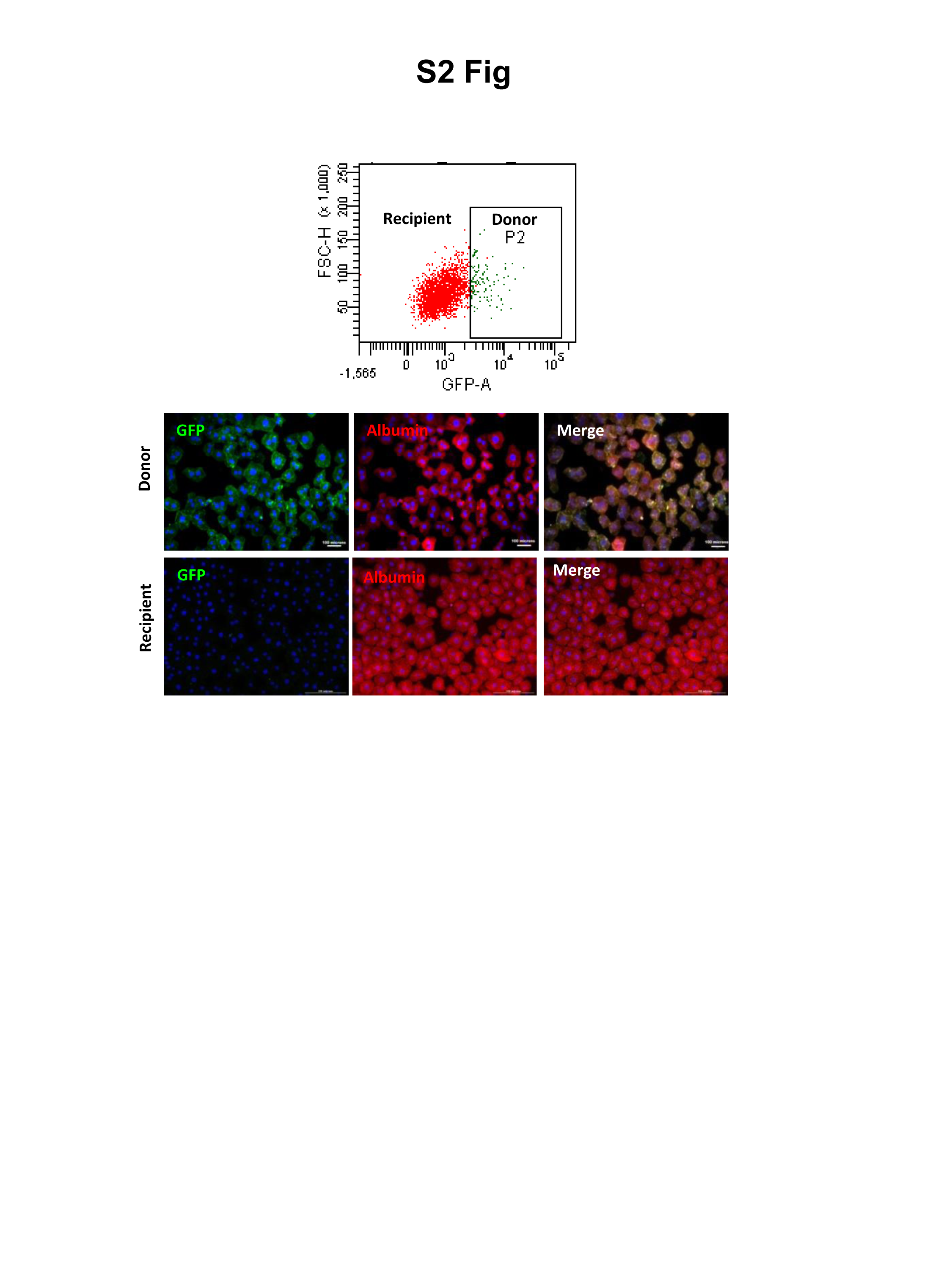

Supplement: S2 Fig — (A) Single cell suspension of hepatocytes was made by two-step collegenase perfusion and the donor BM-derived hepatocytes were isolated by flow cytometry on the basis of eGFP marker. (B) The sorted cells were stained for GFP and albumin markers (anti-GFP/donkey anti-mouse Alexa fluor 488 and anti-albumin /donkey anti-goat Alexa fluor 594) to further analyze the purity of post sort samples. (TIF) [file pone.0173977.s002.tif]

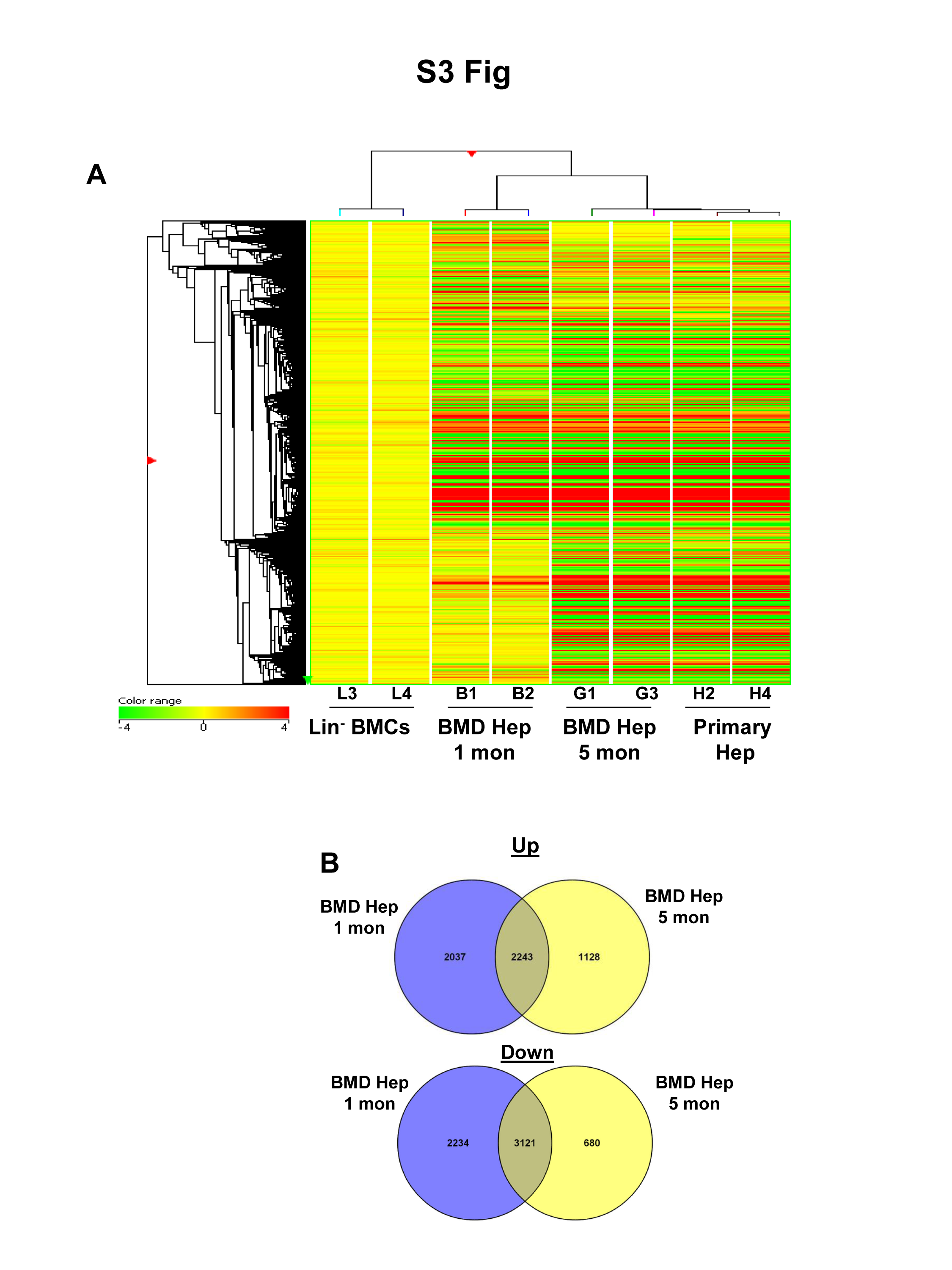

Supplement: S3 Fig — (A) Hierarchical clustering of differentially regulated genes in BM derived hepatocytes– 1 month (B1, B2) and 5 month (G1, G3), primary hepatocytes (H2, H4) with respect to control–Lin- BMCs (L3, L4). (B) Venn diagram illustrating the overlap in differential gene expression profile between BM–derived hepatocytes after 1 month and 5 months of transplantation relative to Lin- BMCs. (TIF) [file pone.0173977.s003.tif]

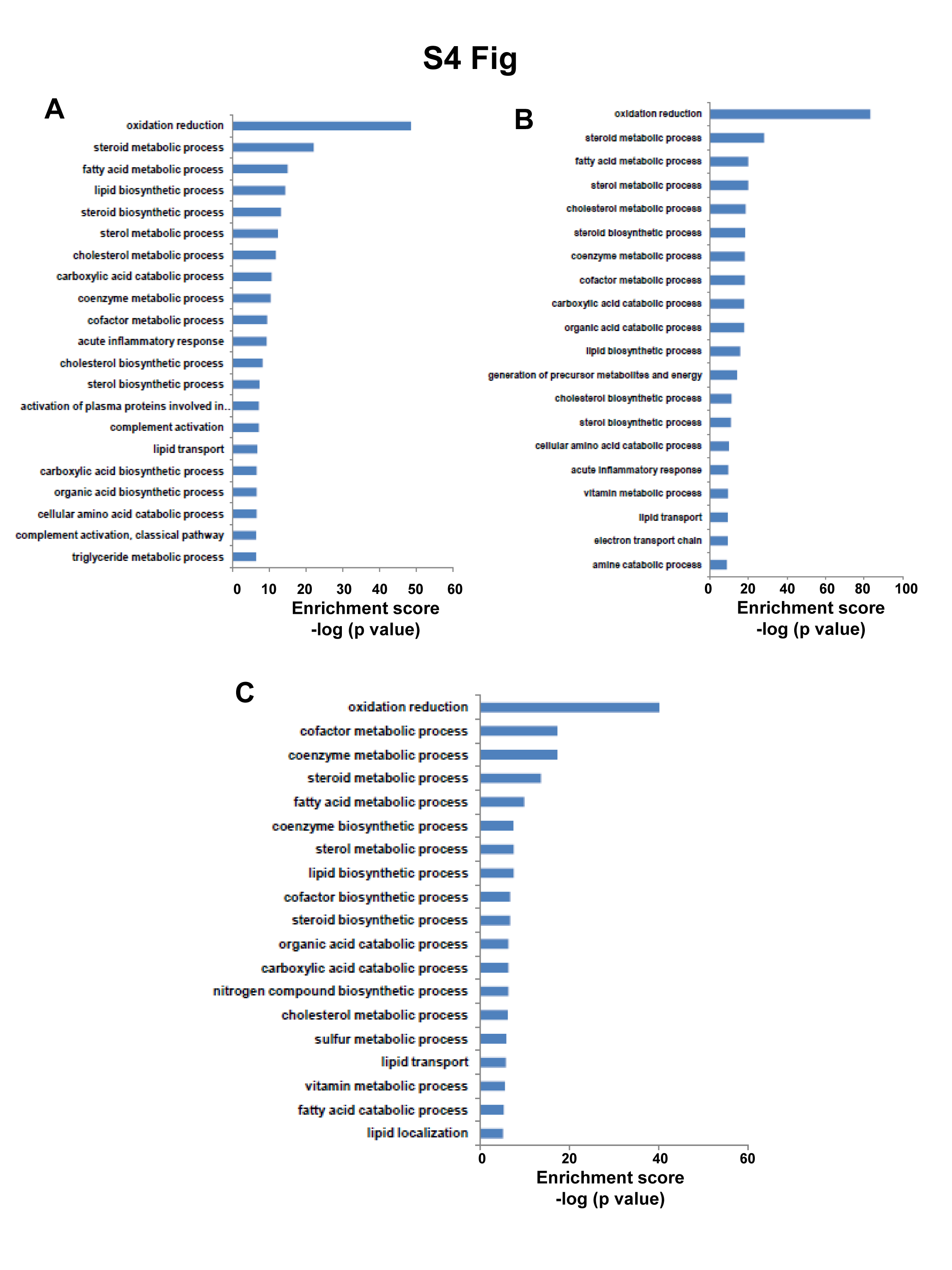

Supplement: S4 Fig — Gene ontology analysis of the total up-regulated genes in BM derived hepatocytes after (A) 1 month and (B) 5 months of transplantation with respect to Lin- BM cells. (C) Gene ontology analysis of the commonly up-regulated genes in BM derived hepatocytes after 1 and 5 months of transplantation with respect to Lin- BM cells. Number of experiment (n) = 2. (TIF) [file pone.0173977.s004.tif]

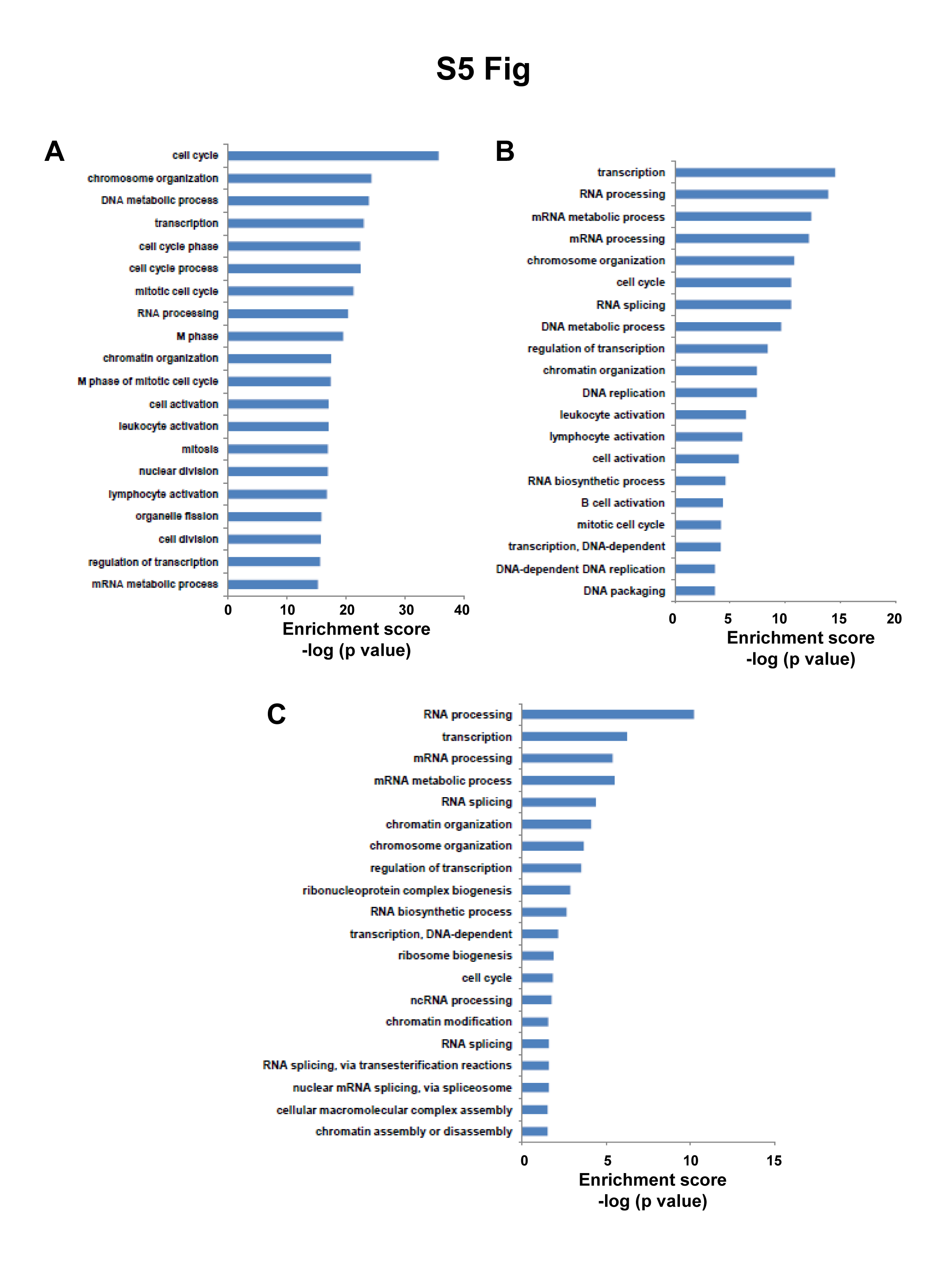

Supplement: S5 Fig — Gene ontology analysis of the total down-regulated genes in BM derived hepatocytes after (A) 1 month and (B) 5 months of transplantation with respect to Lin- BM cells. (C) Gene ontology analysis of the commonly down-regulated genes in BM derived hepatocytes after 1 and 5 months of transplantation with respect to Lin- BM cells. Number of experiment (n) = 2. (TIF) [file pone.0173977.s005.tif]

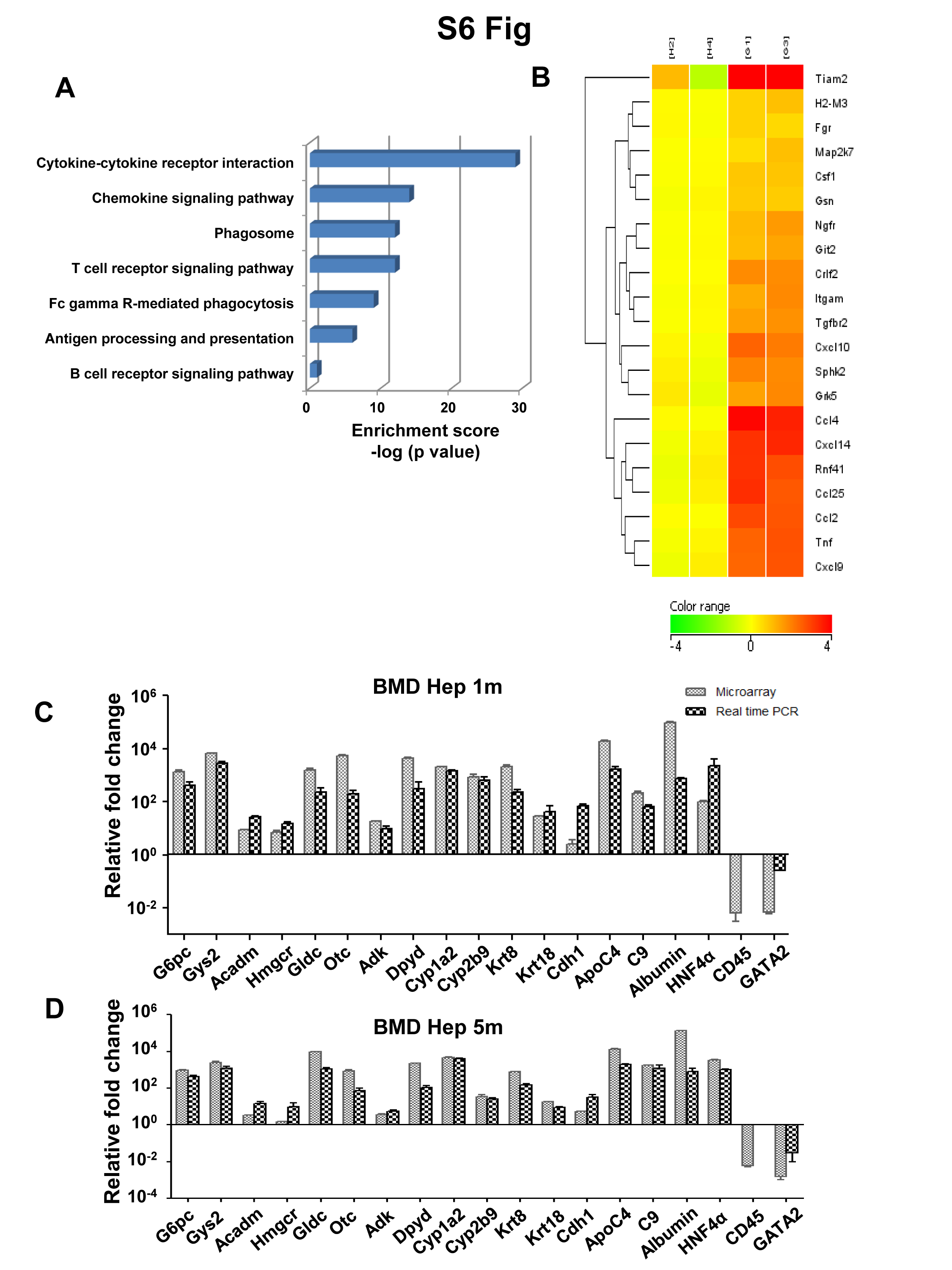

Supplement: S6 Fig — (A) Functional annotation of these genes obtained by DAVID Bioinformatics Resources 6.7. Number of experiment (n) = 2. (B) Heat map of hematopoietic genes, the expression of which is retained in donor derived hepatocytes even after 5 months of transplantation. Number of experiment (n) = 2. (C & D) Fold change in expression of few specific hepatic genes in BM-derived hepatocytes relative to Lin- BM cells after 1 and 5 months of transplantation. Number of experiment (n) = 3. (TIF) [file pone.0173977.s006.tif]

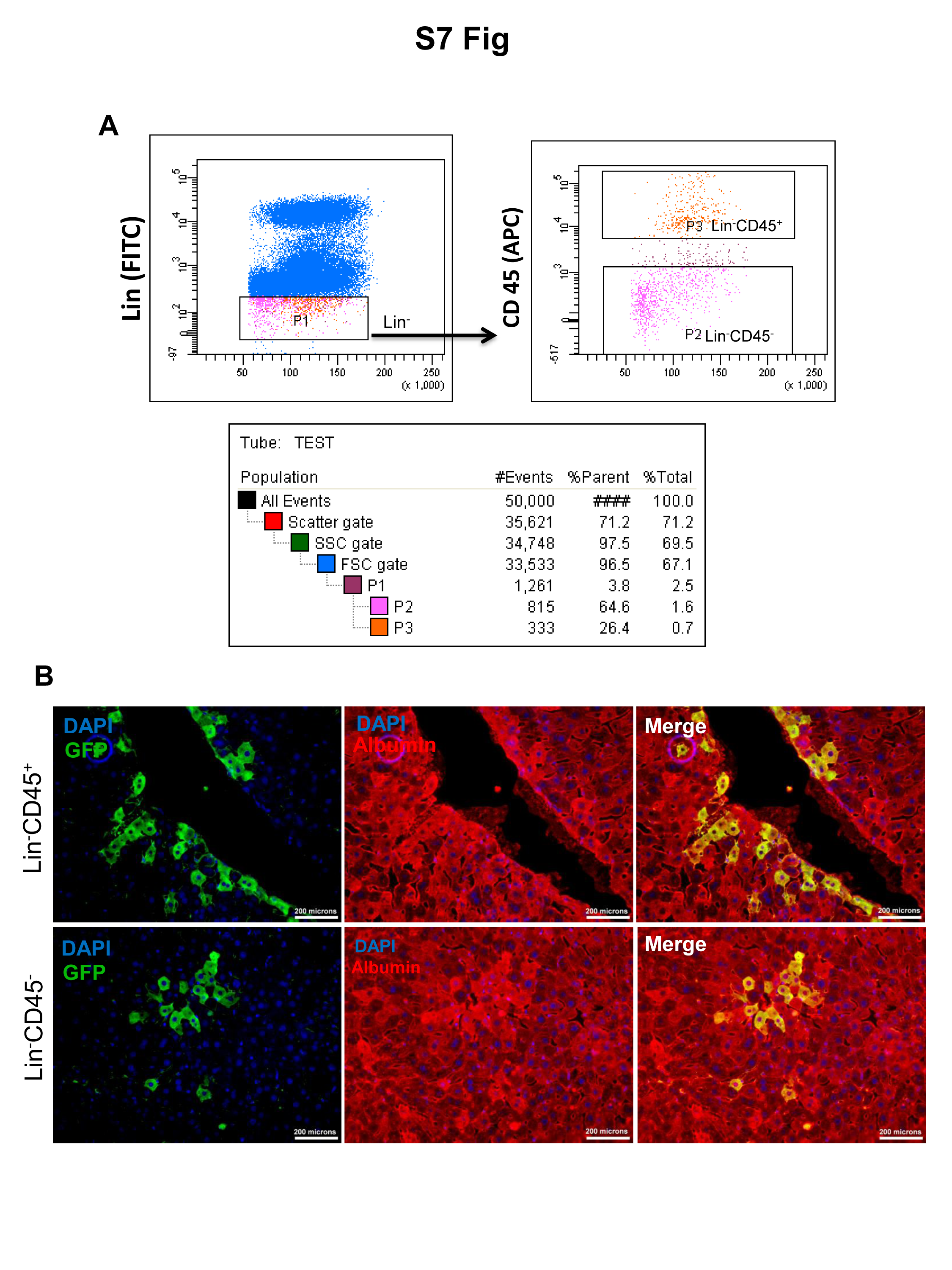

Supplement: S7 Fig — (A) Flow cytometric analysis for CD45+ and CD45- fractions of cells present in Lin- BMCs. (B) Engraftment of Lin-CD45+ and Lin-CD45- fractions of Lin- cells in damaged liver of mice after 1 month of transplantation and albumin expression by the engrafted cells. Number of mice per group = 3. (TIF) [file pone.0173977.s007.tif]

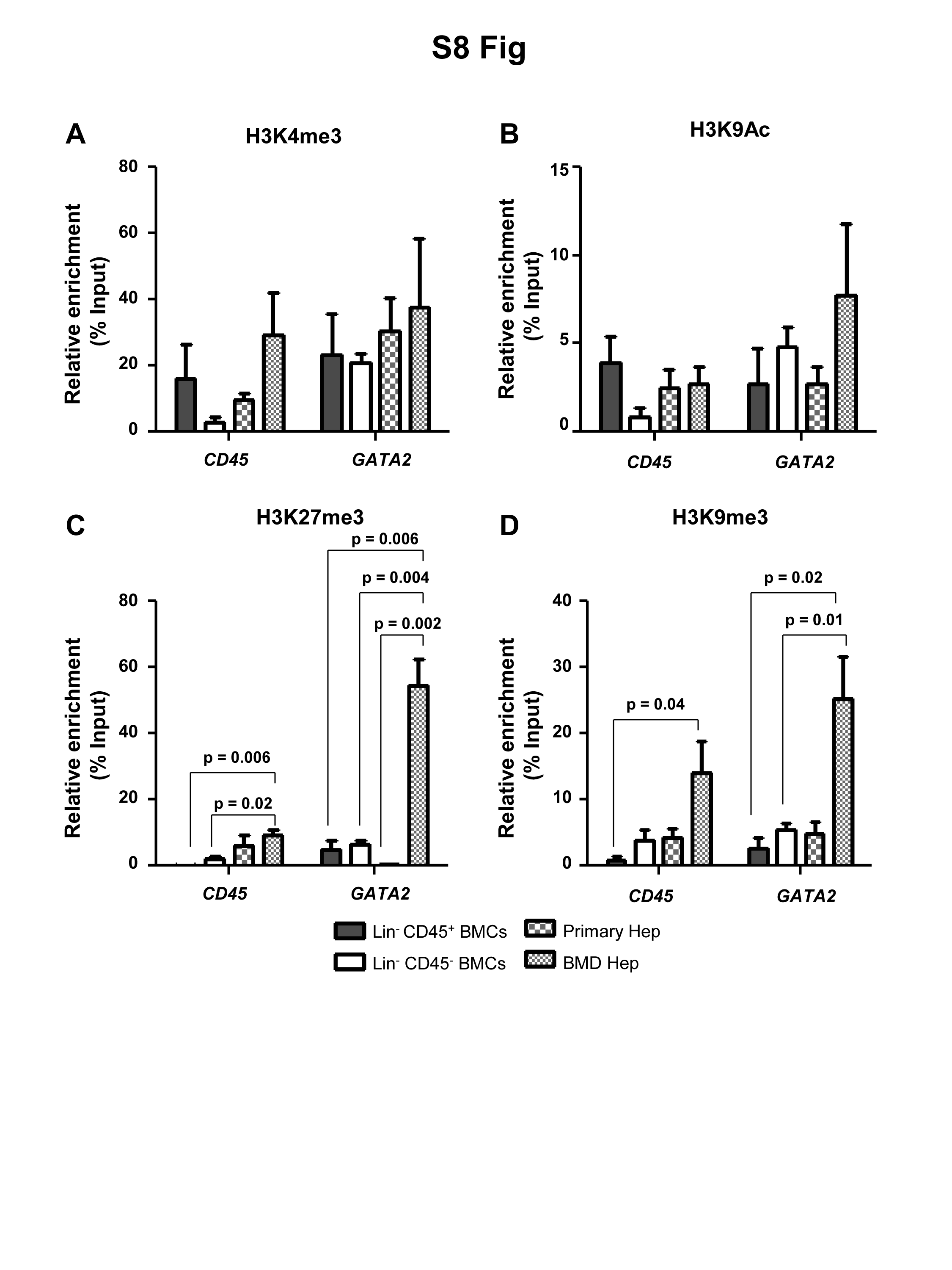

Supplement: S8 Fig — BM-derived hepatocytes are isolated after 5 months of transplantation for ChIP-qPCR analysis. Lin-CD45+ and Lin-CD45- BM cells served as negative controls and primary hepatocytes as positive control. ChIP-qPCR analyses of (A) H3K4me3 (B) H3K9Ac (C) H3K27me3 and (D) H3K9me3 at the promoters of hematopoietic genes in BM-derived hepatocytes. Enrichment of the marks in the immuno-precipitated samples over input samples has been calculated. Number of experiment (n) = 3. S1 Table for IgG controls. (TIF) [file pone.0173977.s008.tif]

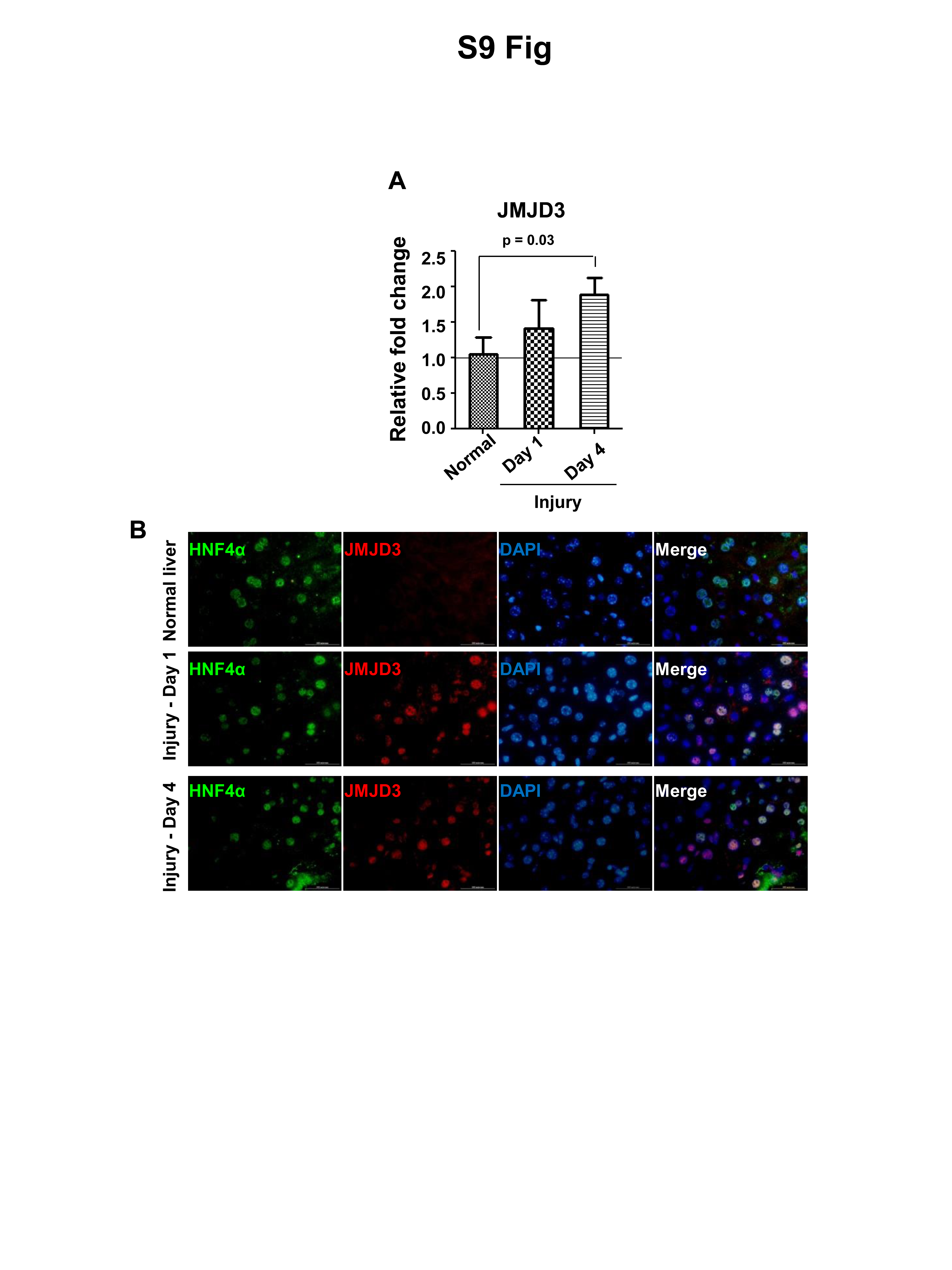

Supplement: S9 Fig — (A) Expression of JMJD3 in livers harvested day 1 and day 4 post induction of injury by acetaminophen relative to that of primary hepatocytes. (B) Immuno-histochemical analysis of liver cryo-sections to study expression of JMJD3 in hepatocytes after liver injury (scale = 100μm, 600X magnification). p value < 0.05 was considered as significant change. Number of animals used for analysis in each group = 5. Data are represented as mean ± SEM. (TIF) [file pone.0173977.s009.tif]

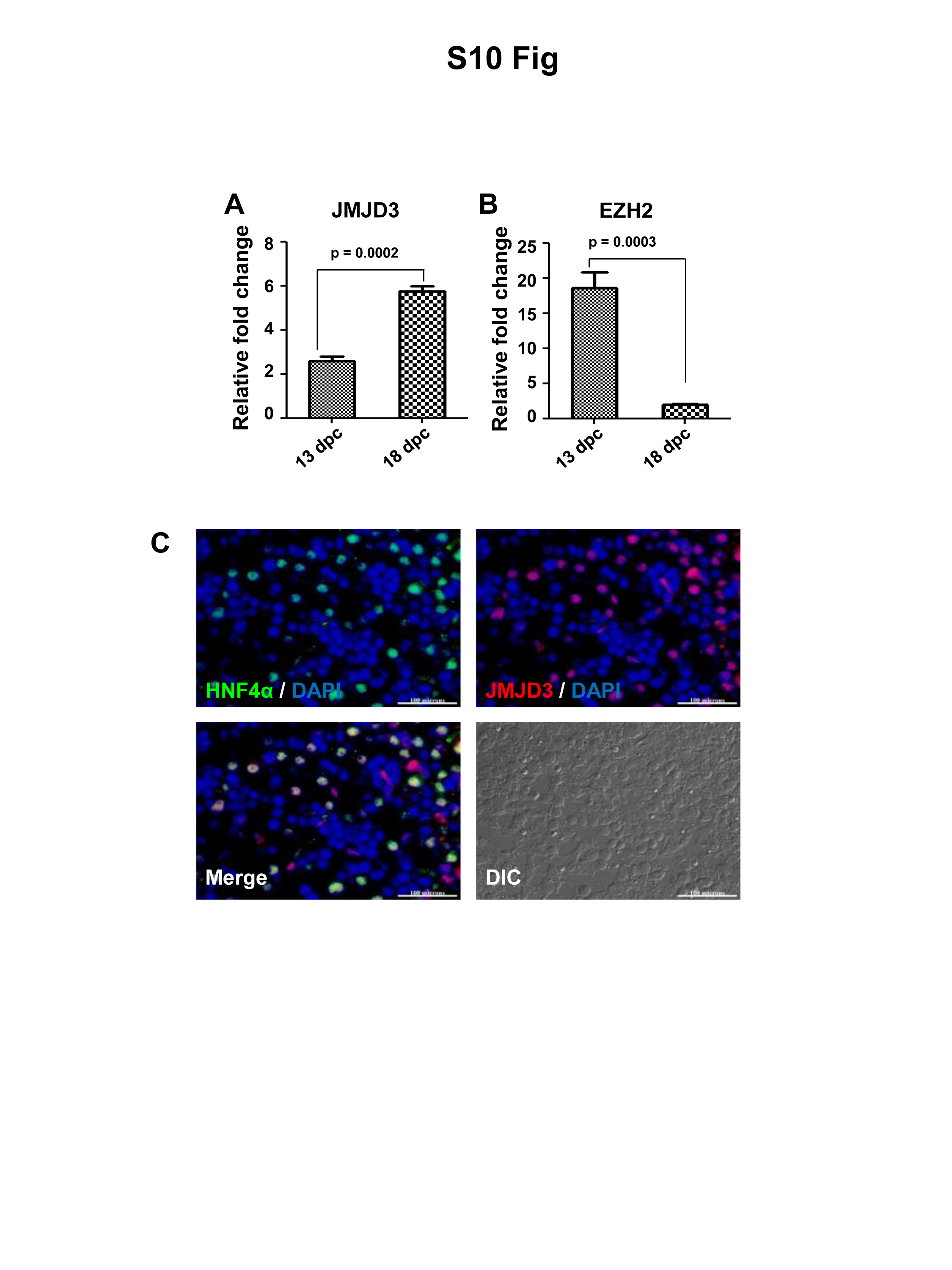

Supplement: S10 Fig — (A) Expression of JMJD3 in fetal livers of 13 and 18dpc mouse embryos relative to expression in normal adult liver. (B) Expression of EZH2 in fetal livers of 13 and 18dpc mouse embryos relative to expression in normal adult liver. (C) Immuno-histochemical analysis of 18dpc fetal liver cryo-sections to study expression of JMJD3 during liver development (scale = 100μm, 600× magnification). Number of mice = 3. (TIF) [file pone.0173977.s010.tif]

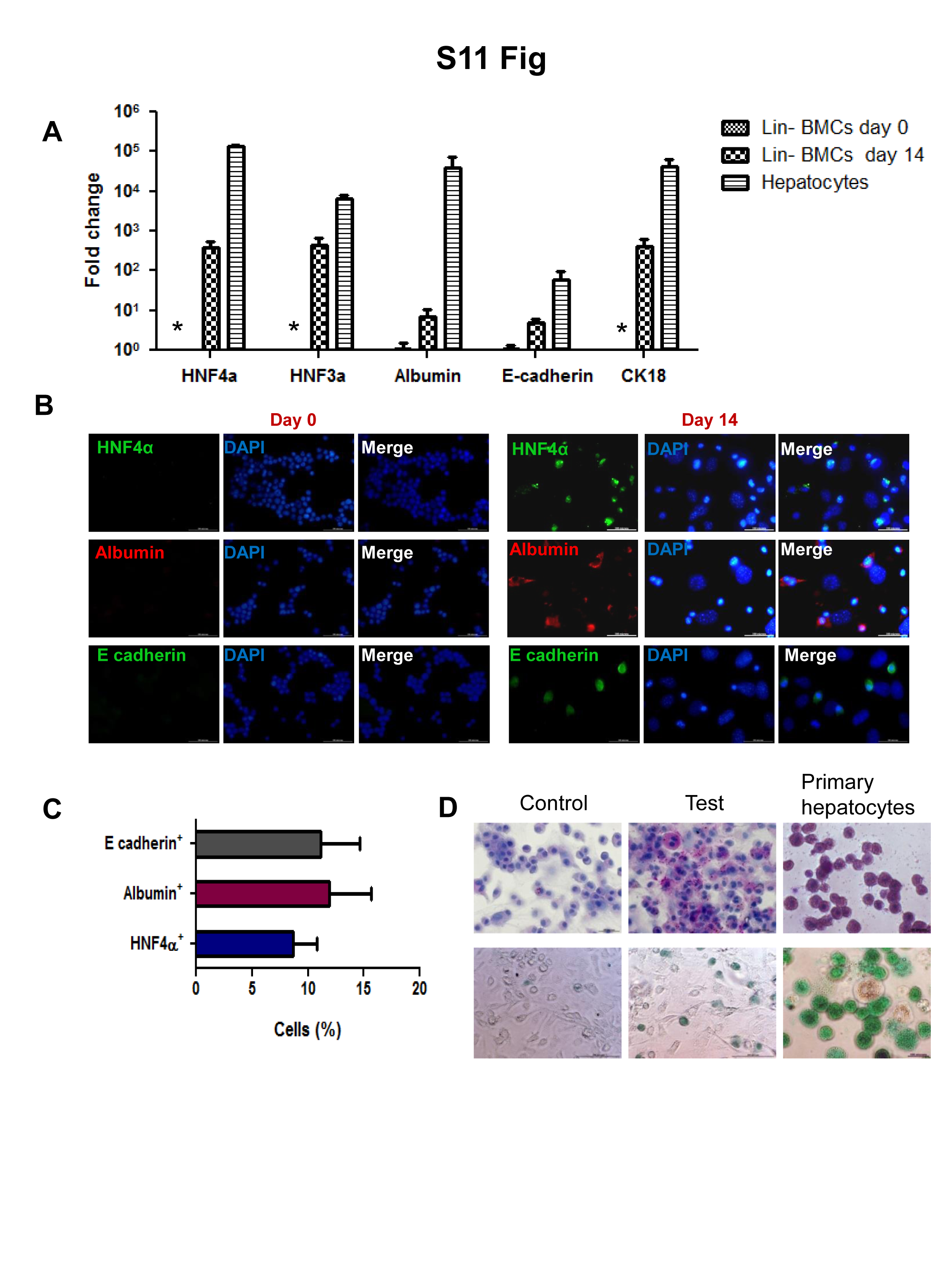

Supplement: S11 Fig — (A) Lin- BMCs were isolated and cultured on plates coated with hyaluronic acid, laminin and collagen I under hepatic differentiation conditions. After 14 days of culture cells were harvested and RNA was isolated. Expression of hepatic markers like HNF4α, HNF3α, albumin, E cadherin and CK18 was determined. Expression levels were calculated relative to Lin- BMCs. *denotes that no Ct was observed in these samples. Number of experiments = 3. (B) Expression of hepatic markers like HNF4α, albumin and E cadherin after 14 days of culture was determined by immuno-cytochemistry (scale = 100μm, 600X magnification). (C) Quantification of in vitro differentiated cells was performed using Image J 1.48 version. Number of experiments = 3. (D) Functional characterization of hepatocytes derived from Lin- BMCs. Periodic acid schiff staining was performed in control Lin- BMCs, test samples in which Lin- BMCs were cultured under hepatic differentiation conditions for 30 days and primary hepatocytes (upper panel) (Scale = 100μm; 600X magnification). Indocyanin green uptake was assessed in these cells after 30 days of culture in control and test with primary hepatocytes as positive control (lower panel) (Scale = 200μm; 600X magnification). Number of experiments = 3. Data are represented as mean ± SEM. (TIF) [file pone.0173977.s011.tif]

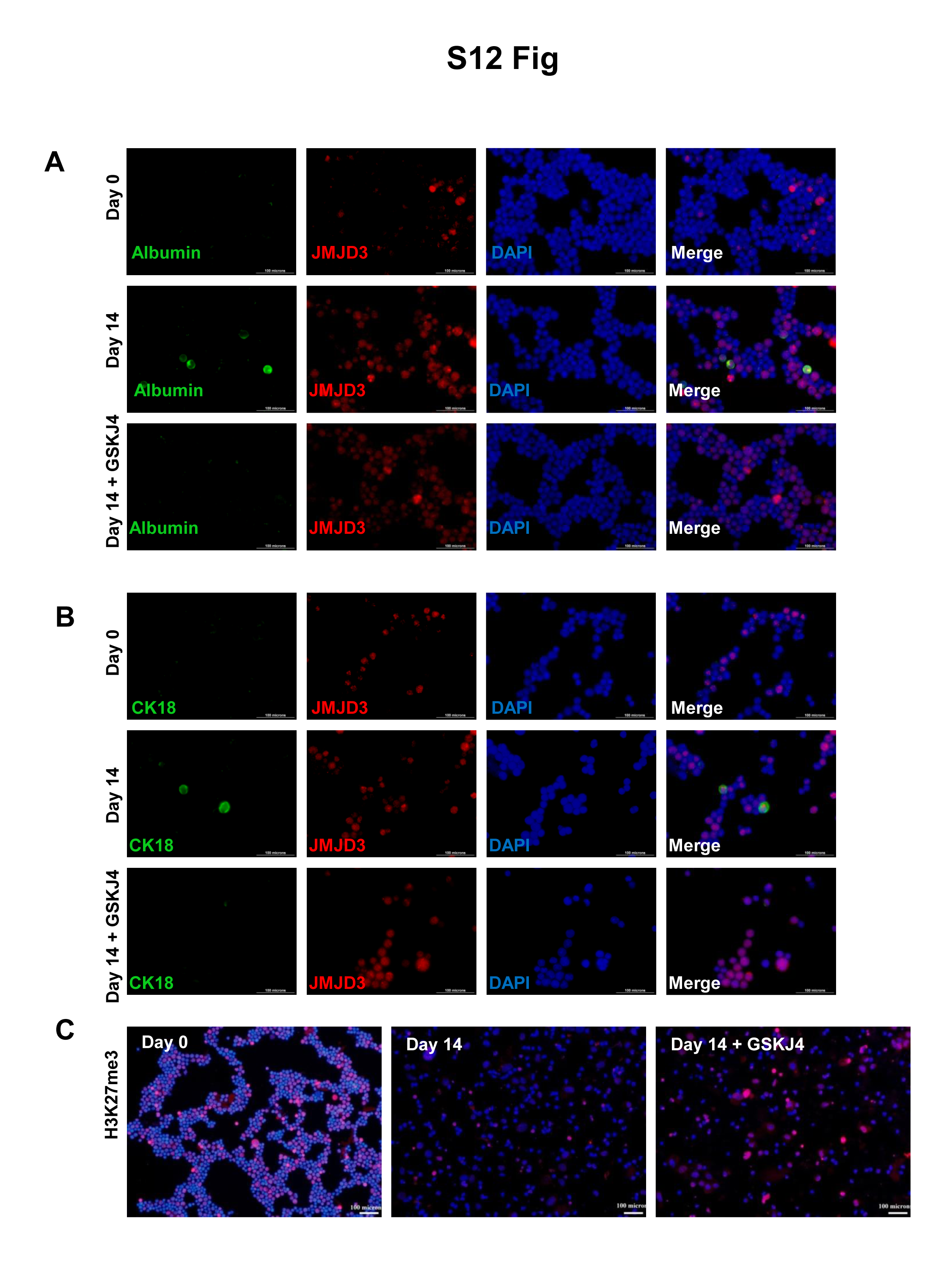

Supplement: S12 Fig — Expression of hepatic markers (A) albumin and (B) CK18 and induction of JMJD3 as analyzed by immuno-cytochemistry in cyto-spun cells after 14 days of culture (scale = 100μm, 600× magnification) in presence and absence of GSKJ4. (C) Change in nuclear levels of H3K27me3 after 14 days of culture of Lin- BMCs under hepatic differentiation conditions with and without GSKJ4 was determined by immuno-cytochemical analysis (Scale = 100μm, 600X magnification). Cyto-spun cells were stained with rabbit anti-H3K27me3/anti-rabbit Alexa Fluor 594. Number of experiments = 3. (TIF) [file pone.0173977.s012.tif]
