# Supplementary material for: JMJD3 aids in reprogramming of bone marrow progenitor cells to hepatic phenotype through epigenetic activation of hepatic transcription factors
Source: PLoS One. 2017 Mar 22;12(3):e0173977. doi: 10.1371/journal.pone.0173977 (PMC5362104; doi:10.1371/journal.pone.0173977)
Supplement: S1 Table — (DOC) [file pone.0173977.s013.doc]

**S1 Table. Primers for analysis of gene expression by real time PCR**

| **Gene** | **Forward primer** | **Reverse Primer** | **Amplicon size (bp)** |
| --- | --- | --- | --- |
| *GAPDH* | ACGGCCGCATCTTCTTGTGCA | CAGGCGCCCAATACGGCCAA | 100 |
| *GATA4* | AGGAGGGGATTCAAACCAGA | CATTGCTGGAGTTACCGCT | 123 |
| *GATA6* | TTTTATTCACCAGCAGCGAC | CGCTACTCCAACCTGACTTTT | 147 |
| *HNF3α* | GATGTTAGGGACTGTGAAGA | GTTCATGTTGCTGACAGG | 106 |
| *HNF3β* | GCGAGTTAAAGTATGCTGGG | ATGTTGCTCACGGAAGAGTAG | 102 |
| *HNF1α* | CACCCATGAAGACACAGAAG | CTTCTTAGTTGGCAGCTCAT | 140 |
| *HNF1β* | CCTCTCACCTGACGATAAAA | ATGAGGTTCTGAGATTGCTG | 120 |
| *HNF4α* | GACCCAGCCTACACCA | TGTTGGATGAATTGAGGTTG | 100 |
| *HNF6* | GAGCAAACTCAAGTCGGGT | TTTTTGGGGGTGTTGCCTCT | 150 |
| *OC2* | GTAAACTCAAATCAAATCTGGCAGGGA | TTTCTTCTGCGAGTTGTTCCT | 149 |
| *CEBPα* | GCCATGCCGGGAGAACTCTA | CTCTGGAGGTGACTGCTCATC | 90 |
| *CEBPβ* | CGTTCATGCACCGCCTG | AGTCGGGCTCGTAGTAGTAGAAG | 102 |
| *TDO* | ACTGTCATACCGTGCACTCC | GTGAGGTCAGCAGCTGGAAAG | 92 |
| *Albumin* | TTTTCCAGGGGTGTGTTTCG | TGGGAAAAGGCAATCAGGAC | 110 |
| *CK18* | AAGGTGAAGCTTGAGGCAGA | CTGCACAGTTTGCATGGAGT | 111 |
| *E-cadherin* | GGCGTTTTCATCATTGAGAG | ACACGGCATGAGAATAGAG | 100 |
| *CYP1A2* | CTGACTCCCACAACTCTG | CTAAACAGAAGATGGCAGTG | 118 |
| *CYP2B9* | TCAAGTACCCCCATGTCACAG | TGGTGCGGTCATCAAGAGT | 90 |
| *GATA2* | ATGAATGGACAGAACCGGC | TCGTCTGACAATTTGCACAAC | 92 |
| *CD45* | AAGTGCAGAAACAGAAGATG | CGATGATGTCATAGAGGAAC | 116 |
| *Sca1* | TACCCTGATGGAGTCTGTGT | TTCAATATTAGGAGGGCAGA | 108 |
| *c-Myc* | CTCTCCTTCCTCGGACTCG | GCCTCTTCTCCACAGACACC | 161 |
| *Ctnnb1* | CAGTGCAGGAGGCCGA | GCCATGTCCAACTCCATCAG | 122 |
| *AFP* | GAAAAACTCTGGCGATGGGT | CAGCAGCCTGAGAGTCCATA | 105 |
| *G6Pc* | GCCTATAATAAAGCAGTTCC | GATAGCAAGAGTAGAAGTGA | 115 |
| *Gys2* | TAGTGTTTTTCATCATGCCT | CAAACTTCTCCTTCAAACAA | 114 |
| *Acadm* | AGGGTGACGAGTATGTTAT | GTACTTTAGGATCTGGGTTAG | 105 |
| *Hmgcr* | CAGTTCCTTCCGCCC | CGGAAAAGTCTTGACAACAT | 117 |
| *Gldc* | CATTTTGCGGAACTTACTG | CATGGTCTGGTAGTTGAGTA | 109 |
| *Otc* | GTATTTTATTCTCCACGGTC | GCACAGGTGAGTAGTCT | 103 |
| *Adk* | AGCATCGGACATCAGG | AGAGATGTCAAGAAGAGGA | 113 |
| *Dpyd* | GAAAACAGCTGCATATTGG | ATCCAGTCACTCTTGTTGTA | 108 |
| *Krt8* | ACAGAGATGGAGAATGAATTT | AAGTTGATCTCGTCGGT | 110 |
| *ApoC4* | TTCTTGGTCAGCTTTGTAG | CTCCACCATCTCCAACA | 111 |
| *C9* | TACGAGACGAAAAGACATAC | TTCGTCATAGTTCTCAGTTC | 101 |
| *MLL* | ACGGCCCTGTTGAATTCTCG | GGGGAGCTTCGGGAAGGTAT | 113 |
| *p300* | AATTAAAAATGGCCGAGAATGTGG | GGTAAGTCATGTTCCAGGTCAA | 139 |
| *SETDB1* | GCAACTCAGAACCCGTCCTA | ATAGGCTGTAGGGGCTCCAT | 120 |
| *UTX* | TCTGGAAAATTTTGTGGTGCT | AGATGAGGCGGATGGTAATG | 117 |
| *JMJD3* | CACCCCACTTCTGCTGTAA | AGAAAGCCAATCATCACCCT | 112 |
| *EZH2* | CCAAATCTGTTCAGAGGGAGCA | TGTTGGGTGTTGCATGGAAGG | 123 |
| *HGF* | TTATGTGCTGGGGCTGAAAA | GACCAGGAACAATGACACCA | 115 |
| *OSM* | CGGCTTCTAAGAACACTGCT | TGAGGAGCTGAGAGGAAGAG | 100 |
| *SDF1α* | TGTGCCCTTCAGATTGT | GGGCTGTTGTGCTTAC | 120 |
| *TGFα* | GGTATCCTGTTAGCTGTGTG | GTACTGAGTGTGGGAATCTG | 120 |
| *FGF1* | AAGGGCTTTTATACGGCT | TTTCCAGTTCTTCTCCGCAT | 100 |
| *Flt3L* | CTGTTGCTGCTGCTGAGT | AAGCAGGTGGTCAGTCAA | 113 |
| *VEGFα* | CCTTCGTCCTCTCCTTACCC | AAGCCACTCACACACACAGC | 117 |
| *IL6* | GAAATGATGGATGCTACC | GGCTTTGTCTTTCTTGTT | 133 |
| *SCF* | TGTTCTTGCTACCCGTGACCT | CCTCCAGAGTCCTGCTCCG | 122 |
