# Supplementary material for: JMJD3 aids in reprogramming of bone marrow progenitor cells to hepatic phenotype through epigenetic activation of hepatic transcription factors
Source: PLoS One. 2017 Mar 22;12(3):e0173977. doi: 10.1371/journal.pone.0173977 (PMC5362104; doi:10.1371/journal.pone.0173977)
Supplement: S2 Table — (DOC) [file pone.0173977.s014.doc]

**S2 Table. Primers for analysis promoters of ChIP DNA**

| **Gene** | **Forward primer (5’-3’)** | **Reverse Primer (5’-3’)** | **Amplicon size (bp)** | **Region analyzed** |
| --- | --- | --- | --- | --- |
| *HNF4α* | AAAGCATGACAATCCCCAGC | CCCGCCCGGTTATCTTATTG | 105 | -127 to -23 |
| *CEBPα* | GGAAAGTCACAGGAGAAGGC | GCTTTTATAGAGGGTCGGGC | 113 | -143 to -20 |
| *HNF1α* | TGACCCACTCCAGGAAATGT | CCACTCTCCAGGATGCTTTC | 139 | -373 to -234 |
| *HNF3α* | CTCGGGCTTTGTAGGT | AGTTGAGCTGATGTGGAT | 100 | -123 to -23 |
| *CEBPβ* | ACCAGGCACACCAAG | CTCATGTTTGGAGGAACC | 103 | -142 to -39 |
| *HNF6* | CTAGCAACTCAGACTCAGGG | CTTCTTCACTACCTCCCTCT | 88 | -374 to -286 |
| *HNF3β* | CCTGGCTTCCTCTCACCTAA | TGAGCGGCGTGTGATATAG | 88 | -561 to -473 |
| *GATA4* | TACATAATCTCCTGGGCTGG | AAAACCTGAACTGGCCCTTA | 98 | -611 to -513 |
| *GATA2* | CTGACTAGTGGCCCAAGC | GGGGGAGGGAACGCC | 101 | -321 to -220 |
| *CD45* | TGATGACTTGGGAGGGATGT | TATGCTGTTAGCGGTTCGTC | 129 | -344 to -215 |
