# Supplementary material for: JMJD3 aids in reprogramming of bone marrow progenitor cells to hepatic phenotype through epigenetic activation of hepatic transcription factors
Source: PLoS One. 2017 Mar 22;12(3):e0173977. doi: 10.1371/journal.pone.0173977 (PMC5362104; doi:10.1371/journal.pone.0173977)
Supplement: S3 Table — (DOC) [file pone.0173977.s015.doc]

**S3 Table. Percentage enrichment of input in rabbit IgG controls for ChIP-qPCR analysis** **of histone marks**

| **Gene Promoters** | **Lin^-^CD45^+^ BMCs** | **Lin^-^CD45^-^ BMCs** | **Primary Hepatocytes** | **BM-derived Hepatocytes** |
| --- | --- | --- | --- | --- |
| *HNF4α* | 0.05 + 0.05 | 0.69 + 0.63 | 1.09 + 1.00 | 0.69 + 0.57 |
| *CEBPα* | 0.00 + 0.00 | 0.00 + 0.00 | 0.03 + 0.03 | 0.00 + 0.00 |
| *HNF1α* | 0.06 + 0.06 | 0.04 + 0.04 | 0.50 + 0.44 | 0.07 + 0.07 |
| *HNF3α* | 0.05 + 0.05 | 0.17 + 0.17 | 0.35 + 0.33 | 0.03 + 0.03 |
| *CEBPβ* | 0.06 + 0.06 | 0.18 + 0.18 | 1.23 + 1.16 | 0.24 + 0.14 |
| *HNF6* | 0.07 + 0.04 | 0.47 + 0.26 | 1.35 + 1.27 | 0.11 + 0.06 |
| *HNF3β* | 0.08 + 0.07 | 0.61 + 0.49 | 0.71 + 0.68 | 0.00 + 0.00 |
| *GATA4* | 0.07 + 0.07 | 0.27 + 0.15 | 0.64 + 0.58 | 0.11 + 0.07 |
| *CD45* | 0.00 + 0.00 | 0.32 + 0.28 | 0.82 + 0.72 | 0.95 + 0.75 |
| *GATA2* | 0.14 + 0.14 | 0.00 + 0.00 | 0.44 + 0.37 | 0.10 + 0.10 |
