# Supplementary material for: JMJD3 aids in reprogramming of bone marrow progenitor cells to hepatic phenotype through epigenetic activation of hepatic transcription factors
Source: PLoS One. 2017 Mar 22;12(3):e0173977. doi: 10.1371/journal.pone.0173977 (PMC5362104; doi:10.1371/journal.pone.0173977)
Supplement: S4 Table — (DOC) [file pone.0173977.s016.doc]

**S4 Table. Percentage enrichment of input in rabbit IgG controls for ChIP-qPCR analysis of binding of EZH2 and JMJD3 to gene promoters**

| **Gene Promoters** | **Lin^-^CD45^+^ BMCs** | **Lin^-^CD45^-^ BMCs** | **Primary Hepatocytes** | **BM-derived Hepatocytes** |
| --- | --- | --- | --- | --- |
| *HNF4α* | 0.03 + 0.01 | 0.16 + 0.15 | 0.00 + 0.00 | 0.01 + 0.01 |
| *CEBPα* | 0.01 + 0.00 | 0.09 + 0.09 | 0.02 + 0.02 | 0.01 + 0.01 |
| *HNF1α* | 0.02 + 0.00 | 0.05 + 0.02 | 0.00 + 0.00 | 0.00 + 0.00 |
| *HNF3α* | 0.01 + 0.01 | 0.07 + 0.07 | 0.00 + 0.00 | 0.00 + 0.00 |
| *CEBPβ* | 0.01 + 0.00 | 0.03 + 0.02 | 0.00 + 0.00 | 0.02 + 0.01 |
| *HNF6* | 0.04 + 0.01 | 0.13 + 0.07 | 0.00 + 0.00 | 0.03 + 0.00 |
| *HNF3β* | 0.01 + 0.00 | 0.03 + 0.01 | 0.00 + 0.00 | 0.01 + 0.00 |
| *GATA4* | 0.05 + 0.04 | 0.06 + 0.01 | 0.01 + 0.01 | 0.00 + 0.00 |
| *CD45* | 0.03 + 0.02 | 0.01 + 0.01 | 0.07 + 0.04 | 0.00 + 0.00 |
| *GATA2* | 0.00 + 0.00 | 0.12 + 0.03 | 0.00 + 0.00 | 0.02 + 0.01 |
