# Supplementary material for: JMJD3 aids in reprogramming of bone marrow progenitor cells to hepatic phenotype through epigenetic activation of hepatic transcription factors
Source: PLoS One. 2017 Mar 22;12(3):e0173977. doi: 10.1371/journal.pone.0173977 (PMC5362104; doi:10.1371/journal.pone.0173977)
Supplement: S5 Table — (DOCX) [file pone.0173977.s017.docx]

**S5 Table. Percentage Enrichment of input in rabbit IgG controls for ChIP-qPCR analysis of binding of JMJD3 and H3K27me3 to gene promoters**

| **Gene Promoters** | **Day 0** | **Day 14** | **Day 14 + GSKJ4** |
| --- | --- | --- | --- |
| *HNF4α* | 0.29+0.16 | 0.58+ 0.08 | 0.11+ 0.06 |
| *HNF3α* | 0.11+0.11 | 0.59+ 0.03 | 0.40+0.20 |
| *HNF3β* | 0.22+0.11 | 0.70+ 0.07 | 0.18+0.10 |
| *HNF1α* | 0.27+0.12 | 0.66+0.25 | 0.32+0.16 |
| *CEBPα* | 0.18+0.13 | 0.12+0.06 | 0.19+0.09 |
| *GATA4* | 0.18+0.15 | 0.61+0.13 | 0.36+0.21 |
